# Human Induced Pluripotent Stem Cell-Derived Cardiomyocytes, in Contrast to Adipose Tissue-Derived Stromal Cells, Efficiently Improve Heart Function in Murine Model of Myocardial Infarction

**DOI:** 10.3390/biomedicines8120578

**Published:** 2020-12-07

**Authors:** Jacek Stępniewski, Mateusz Tomczyk, Kalina Andrysiak, Izabela Kraszewska, Alicja Martyniak, Agnieszka Langrzyk, Klaudia Kulik, Ewa Wiśniewska, Mateusz Jeż, Urszula Florczyk-Soluch, Katarzyna Polak, Paulina Podkalicka, Neli Kachamakova-Trojanowska, Alicja Józkowicz, Agnieszka Jaźwa-Kusior, Józef Dulak

**Affiliations:** 1 Department of Medical Biotechnology, Faculty of Biochemistry, Biophysics and Biotechnology, Jagiellonian University, Gronostajowa 7, 30-387 Kraków, Poland; tomczyk.mateusz@gmail.com (M.T.); kalina.andrysiak@doctoral.uj.edu.pl (K.A.); izabela.kraszewska@doctoral.uj.edu.pl (I.K.); alicja.martyniak@doctoral.uj.edu.pl (A.M.); agnieszka.langrzyk@gmail.com (A.L.); klaudiakulik90@gmail.com (K.K.); ewa.wisniewska1987@gmail.com (E.W.); mateusz.jez@doctoral.uj.edu.pl (M.J.); urszula.florczyk@uj.edu.pl (U.F.-S.); kate.polak@uj.edu.pl (K.P.); paulina.podkalicka@doctoral.uj.edu.pl (P.P.); alicja.jozkowicz@uj.edu.pl (A.J.); agnieszka.jazwa@uj.edu.pl (A.J.-K.); 2 Kardio-Med Silesia, Curie-Skłodowskiej 10C, 41-800 Zabrze, Poland; 3 Małopolska Centre of Biotechnology, Jagiellonian University, Gronostajowa 7A, 30-387 Kraków, Poland; neli.kachamakova-trojanowska@uj.edu.pl

**Keywords:** adipose-derived stromal cells, human induced pluripotent stem cells, myocardial infarction, heme oxygenase-1, stromal cell-derived factor-1α

## Abstract

Cell therapies are extensively tested to restore heart function after myocardial infarction (MI). Survival of any cell type after intracardiac administration, however, may be limited due to unfavorable conditions of damaged tissue. Therefore, the aim of this study was to evaluate the therapeutic effect of adipose-derived stromal cells (ADSCs) and human induced pluripotent stem cell-derived cardiomyocytes (hiPSC-CMs) overexpressing either the proangiogenic SDF-1α or anti-inflammatory heme oxygenase-1 (HO-1) in a murine model of MI. ADSCs and hiPSCs were transduced with lentiviral vectors encoding luciferase (Luc), GFP and either HO-1 or SDF-1α. hiPSCs were then differentiated to hiPSC-CMs using small molecules modulating the WNT pathway. Genetically modified ADSCs were firstly administered via intracardiac injection after MI induction in *Nude* mice. Next, ADSCs-Luc-GFP and genetically modified hiPSC-CMs were injected into the hearts of the more receptive *NOD/SCID* strain to compare the therapeutic effect of both cell types. Ultrasonography, performed on days 7, 14, 28 and 42, revealed a significant decrease of left ventricular ejection fraction (LVEF) in all MI-induced groups. No improvement of LVEF was observed in ADSC-treated *Nude* and *NOD/SCID* mice. In contrast, administration of hiPSC-CMs resulted in a substantial increase of LVEF, occurring between 28 and 42 days after MI, and decreased fibrosis, regardless of genetic modification. Importantly, bioluminescence analysis, as well as immunofluorescent staining, confirmed the presence of hiPSC-CMs in murine tissue. Interestingly, the luminescence signal was strongest in hearts treated with hiPSC-CMs overexpressing HO-1. Performed experiments demonstrate that hiPSC-CMs, unlike ADSCs, are effective in improving heart function after MI. Additionally, long-term evaluation of heart function seems to be crucial for proper assessment of the effect of cell administration.

## 1. Introduction

The adult human heart has a minimal ability to regenerate, and thus the loss of viable cardiomyocytes in coronary heart disease, accelerated by myocardial infarction (MI), frequently leads to heart failure (HF). Bergmann et al. reported that cardiomyocyte renewal in physiological conditions in humans is as little as 1% of cells renewed per year at the age of 20 and further drops to 0.4% at the age of 75 [1]. This rate can increase upon myocardial damage; however, no more than 3% of cardiomyocytes located near the injury region activate cell division, which is far too little for meaningful regeneration [2]. Thus, a fibrotic scar produced by fibroblasts and myofibroblasts replaces lost cardiomyocytes, which sustains organ architecture but concurrently impairs the proper electromechanical activity of the heart [3]. Importantly, no available treatment targeting MI enables restoration of functional cardiomyocytes in place of fibrous tissue, and in consequence, heart transplant remains the only therapeutic approach for the most severe cases. Thus, there is an immense need for novel therapies, which would allow for the recovery of viable myocardium in the injured site.

Mesenchymal stromal cells (MSCs), isolated from different tissues including bone marrow, fat and umbilical cord have been extensively investigated in recent years as a novel therapeutic approach for heart regeneration [4]. None of these MSCs, however, demonstrate the ability to differentiate into cardiomyocytes and replace cells lost during MI. Hence, the putative beneficial effect of their administration in animal models has been ascribed to the paracrine activity of these cells [4]. Additionally, as many experiments revealed, most MSCs injected intravenously are trapped in the lungs and rapidly die as a result of complement activation [5,6]. Thus, there is no compelling evidence for their ability to migrate and home into injured tissues upon systemic administration. Similarly, the limited survival of MSCs after local delivery into infarcted myocardium has so far precluded the development of effective therapy for heart damage [7].

In contrast, human induced pluripotent stem cells (hiPSCs), generated from easily accessible somatic cells such as fibroblasts and peripheral blood mononuclear cells (PBMCs) demonstrate the capacity to efficiently differentiate into cardiomyocytes (hiPSC-CMs). Due to such properties, hiPSCs have provided novel opportunities to obtain cells applicable in regenerative medicine [8]. Indeed, preclinical studies confirmed that administration of hiPSC-CMs into the infarcted myocardium improves heart function; however, further investigation is needed to develop the most effective approach for myocardial regeneration upon MI [9]. Importantly, the limited survival of administered cells in the unfavorable environment of damaged tissue represents a major hindrance for successful cell therapy. Thus, novel strategies providing cytoprotection and increased angiogenesis in the site of injection may augment the therapeutic potential of any tested cell types.

Heme oxygenase-1 (HO-1, encoded by *HMOX1*), is the enzyme that catalyzes the reaction of heme degradation leading to the generation of carbon monoxide (CO), iron ions and biliverdin [10]. Studies of our group and many others reported a potent antioxidative, antiapoptotic and anti-inflammatory activity of HO-1 mediated by the products of its activity [11], underlining the cytoprotective role of HO-1 in the maintenance of cellular homeostasis in various stress conditions including oxidative stress and hypoxia. Of note, it was demonstrated that HO-1 promotes cardiac mitochondrial biogenesis and its overexpression protects from doxorubicin-mediated induction of dilated cardiomyopathy through the regulation of mitochondrial quality control in the heart [12]. Our recent study further indicated that lack of HO-1 negatively affects cardiac healing as we observed higher macrophage infiltration, prolonged postischemic inflammatory response and adverse late left ventricle remodeling in HO-1-deficient mice upon induction of MI [13]. Similarly, stromal cell-derived factor 1α (SDF-1α, encoded by *CXCL12*), a potent proangiogenic chemokine, has emerged as an important cardioprotective factor [14]. Particularly, it protects cardiomyocytes from cell death during the first 72 h after left anterior descending artery (LAD) ligation in a murine model of MI and stimulates angiogenesis in hypoxic regions through upregulation of vascular endothelial growth factor (VEGF). Additionally, it was demonstrated that SDF-1α activates STAT3-dependent signaling pathways providing protection of heart function in the murine model of hypoxia/reoxygenation cardiac injury [14]. 

Accordingly, the aim of this study was to investigate and compare the therapeutic potential of genetically modified, either HO-1- or SDF-1α-overexpressing, MSCs and hiPSC-CMs in a murine model of acute MI. We utilized adipose-derived stromal cells (ADSCs) in the study, as adipose tissue represents one of the most convenient cell sources for autologous cell therapy due to its accessibility and ease of isolation [4]. Nevertheless, our results demonstrate no effect of ADSC administration on heart function in sharp contrast to hiPSC-CMs, which provided significant recovery of left ventricle ejection fraction (LVEF) 6 weeks after MI induction. Additionally, our data indicate the importance of long-term evaluation of cell therapy effects, as the most pronounced changes in LVEF occurred between days 28 and 48 of cardiac function analysis, performed after cell administration.

## 2. Methods

### 2.1. ADSC Isolation and Culture

ADSCs were isolated from liposuction aspirate from the Hospital for Minimally Invasive and Reconstructive Surgery in Bielsko-Biała, Poland, upon obtaining informed consent from donors (approval of the Institutional Review Board and Bioethical Committee No. KB/430-62/13) as previously described [15]. The experiments conformed to the principles outlined in the Declaration of Helsinki. Cells were cultured in αMEM medium (Macopharma, Tourcoing, France) supplemented with 10% human platelet lysate (Macopharma), heparin (2 U/mL, Polfa, Warsaw, Poland) and 1% penicillin–streptomycin solution (Sigma-Aldrich, St. Louis, MO, USA) under standard culture conditions (37 °C, 5% CO_2_) and passaged upon reaching full confluency using 0.25% trypsin/EDTA solution (ThermoFisher Scientific, Waltham, MA, USA). Immunophenotyping of these cells confirming expression of CD105, CD29, CD73, CD90 and CD44 surface markers was previously described by Czapla et al. [15], and the same ADSCs were used for transduction and further in vivo administration.

### 2.2. Generation and Culture of hiPSCs

The hiPSC line used in this study was generated from peripheral blood mononuclear cells (PBMCs) isolated from a healthy volunteer upon obtaining informed consent (approval of Jagiellonian University Bioethical Committee No. 122.6120.303.2016; the experiments conformed to the principles outlined in the Declaration of Helsinki). For that purpose, 10 mL of blood was diluted in 30 mL of phosphate-buffered saline (PBS) (Lonza, Basel, Switzerland) with 2 mM EDTA, layered onto 15 mL of Ficoll-Paque (GE Healthcare, Chicago, IL, USA) and centrifuged at 400 × *g* for 40 min at room temperature (RT). The layer of mononuclear cells was then transferred into a new conical tube, washed twice with PBS and resuspended in StemPro-34 medium supplemented with StemPro-34 Nutrient Supplement (ThermoFisher Scientific) and cytokines: 100 ng/mL SCF (Peprotech, Cranbury, NJ, USA), 100 ng/mL FLT3-Ligand (Peprotech), 20 ng/mL IL-3 (Peprotech) and 20 ng/mL IL-6 (PBMC medium). Cells were plated on a 24-well plate and cultured for an additional 6 days with medium changed every other day. Subsequently, 10^5^ isolated PBMCs were reprogrammed using Cytotune-iPS 2.0 Sendai Reprogramming Kit (ThermoFisher Scientific) according to the manufacturer’s protocol. Briefly, cells were suspended in PBMC medium containing KOS (encoding KLF4, OCT4 and SOX2), hc-MYC and hKLF4 Sendai vectors (multiplicity of infection (MOI) = 5, 5 and 3, respectively), centrifuged at 1000 × *g* for 30 min at RT and seeded on a 24-well plate. After 24 h, medium was replaced with fresh PBMC medium for an additional 2 days, after which cells were collected and seeded in 2 wells of a 6-well plate covered with Geltrex (ThermoFisher Scientific, diluted according to manufacturer’s protocol) in StemPro-34 medium supplemented with StemPro-34 Nutrient Supplement. On day 7, half of the medium was replaced with Essential 8 medium (E8, ThermoFisher Scientific), and cells were further cultured in E8 for additional 2 weeks. Then, the hiPSC colonies were picked and expanded. The hiPSC line used in this study was cultured in E8 on Geltrex-coated plates in standard culture conditions (37 °C, 5% CO_2_) and passaged upon reaching 80–90% confluency using 0.5 mM EDTA solution. Pluripotency of hiPSCs was confirmed with immunofluorescent analysis of OCT4, NANOG, SSEA4, TRA-1-60 and TRA-1-81 expression as well as in vitro spontaneous differentiation assay (as described in the Supplementary Methods).

### 2.3. Cardiac Differentiation of hiPSCs

To obtain genetically modified cardiomyocytes, lentiviral vector-transduced and sorted hiPSCs were subjected to cardiac differentiation according to the protocol described by Lian et al. [16]. Briefly, 3 × 10^4^ cells were seeded on Geltrex-coated 24-well plate and cultured in E8 for additional 4 days until they reached 100% confluency. Then, the medium was changed to RPMI1640 (Lonza) supplemented with 2% B27 without insulin (ThermoFisher Scientific, RMPI/B27-ins medium) and 12 µM CHIR99021 (Sigma-Aldrich). After 24 h, the medium was replaced with RMPI/B27-ins for an additional 2 days. On day 3, cells were stimulated with 5 µM IWR-1 (Sigma-Aldrich) in RPMI/B27-ins (collected from the cells and fresh mixed 1:1). After 48 h, the medium was replaced with fresh RPMI/B27-ins; starting from day 7, cells were cultured in RPMI1640 supplemented with 2% B27 (ThermoFisher Scientific, RMPI/B27), which was changed every third day. On day 20, the cells were collected and passaged using Multi Tissue Dissociation Kit 3 (Miltenyi Biotec, Bergisch Gladbach, Germany), collected in RPMI1640 supplemented with 20% FBS (EURx, Gdańsk, Poland), centrifuged (200× *g*, 5 min, RT), resuspended in fresh RPMI/B27 and seeded on Geltrex-coated 10 cm plates.

### 2.4. Experimental Animals

Female, 6–8-week-old athymic *Foxn1^nu^* mice (*Nude*, purchased from Harlan Laboratories, Füllinsdorf, Switzerland) and 6–8-week-old female NOD.CB-17-Prkdc scid/Rj (*NOD/SCID*, Janvier Labs, Le Genest-Saint-Isle, France) mice were used in this study. All animal procedures were performed according to guidelines from Directive 2010/63/EU of the European Parliament on the protection of animals used for scientific purposes and were accepted by the Second Local Ethical Committee for Animal Research in Krakow, Poland (approval numbers: 21/2014, 136/2016 and 58/2018). Mice were maintained under controlled specific pathogen-free (SPF) environmental conditions with standard laboratory food and water provided ad libitum.

### 2.5. Induction of Myocardial Infarction and Administration of Cells

MI was induced in both murine strains as previously described [13]. Briefly, mice were anesthetized with 2,2,2-tribromoethanol (400 mg/kg of body weight), intubated and mechanically ventilated (respiration rate: 220 breaths/min, stroke volume 280 µL). Upon surgical exposition of the heart, LAD was permanently ligated with a silk suture placed 1–2 mm below the left auricle. Instant change of the myocardium color from bright red to white exposed the area of myocardium affected by sudden occlusion of the blood vessel supplying this region. Cells were injected into 4 sites at the border of the ischemic area (as shown in Figure 2a; 2.5 µL containing 1.25 × 10^5^ cells suspended in saline per site; 5 × 10^5^ cells in the volume of 10 µL in total), immediately after LAD ligation. Cells were injected with a 25 μL Hamilton syringe with a 33-gauge needle. MI control group was injected with saline only (without the cells suspended). Sham control mice were subjected to the same procedure; however, upon placing the suture underneath LAD, the vessel was not ligated, and the suture was removed. All animals were subjected to analgesia twice daily for 3 consecutive days after the surgery by subcutaneous injection of buprenorphine at the dose of 0.08 mg/kg of body weight.

Of note, in our research, we have previously used 2,2,2-tribromoethanol to anesthetize mice for MI surgery, and it was very well tolerated by the mice of *C57BL/6xFVB* strain [13], which on the other hand did not tolerate ketamine–xylazine well (unpublished). In that case, all animals anesthetized with 2,2,2-tribromoethanol survived the MI, and any observed demise occurred mainly in wild-type *C57BL/6xFVB* mice between the 3rd and 5th days after LAD ligation due to left ventricular free wall rupture [13]. It is known that ketamine–xylazine produces profound bradycardia [17], and our pilot experiments in *Nude* and *NOD/SCID* mice also revealed that these drugs did not provide satisfactory depth of anesthesia (based on the limb withdrawal response to toe pinch) and their increased doses were lethal (not shown). Therefore, taking into account our previous good experience with 2,2,2-tribromoethanol [13], we used this compound also for anesthesia of *Nude* and *NOD/SCID* mice.

To confirm successful MI induction, 24 h after surgery, facial vein puncture was performed intravitally and peripheral blood (PB) was collected, anticoagulated with heparin (5 U per 1 mL blood) and centrifuged (800 × *g*, 10 min, 4 °C), after which plasma was transferred to a new tube and stored frozen at −80 °C. Cardiac troponin I (cTnI) was assessed in 100 μL of plasma with ELISA (DRG MedTek, Warsaw, Poland) according to the manufacturer’s protocol.

### 2.6. Transthoracic Echocardiography

Transthoracic echocardiography was performed on days 7, 14, 28 and 42 after MI using a Vevo 2100 system (Visual Sonics, Toronto, ON, Canada) with a 30-MHz transducer and inhalation anesthesia (2% isoflurane in air, Aerrane, Baxter, Deerfield, IL, USA) as previously described [13]. The heart was imaged in the bidimensional (2-D) mode, in the parasternal long-axis (PSLAX) view. For analysis of left ventricular end-diastolic volume (LVVd) and left ventricular end-systolic volume (LVVs), the endocardium of the left ventricle was traced at both diastole and systole. An integrated software tool (LV-Trace) was used for single-plane PSLAX analysis.

### 2.7. Bioluminescence Imaging

To assess the presence of ADSCs-Luc-GFP in *Nude* mice, animals were subjected to intracardiac injection of 5 × 10^5^ cells. After 72 h, luciferin solution was administered intraperitoneally (150 mg/kg of body weight in volume of 100 µL) and after 10 min mice were anesthetized by isoflurane inhalation (5% *v/v* isoflurane/air mixture for induction and 1.5–2% *v/v* isoflurane/air for maintenance of anesthesia) and transferred onto a temperature-controlled, heated table (37 °C) in the detecting chamber of an IVIS Lumina II detector. Bioluminescence from the heart was recorded for 5 min. 

To assess the presence of injected genetically modified hiPSC-CMs and ADSCs-Luc-GFP, the *NOD/SCID* mice were subjected to bioluminescence detection using an IVIS Lumina II detector on days 7, 14, 28 and 42 after surgery. For that purpose, luciferin solution was administered intraperitoneally (150 mg/kg of body weight in volume of 100 µL), and after 10 min mice were anesthetized by isoflurane inhalation (5% *v/v* isoflurane/air mixture for induction and 1.5–2% *v/v* isoflurane/air for maintenance of anesthesia), chest-shaved and transferred onto a temperature-controlled, heated table (37 °C) in the detecting chamber. Bioluminescence from the heart was recorded for 10 min. On day 42, the bioluminescence was recorded as described intravitally and directly in hearts isolated from sacrificed animals.

### 2.8. Tissue Collection

On day 42 after MI induction and cell administration, mice were euthanized by intraperitoneal injection of ketamine (200 mg/kg of body weight) and xylazine (40 mg/kg of body weight); their chests were opened and the hearts arrested in diastole by intraventricular injection of 30 mM KCl in saline containing 0.5 U/mL of heparin, followed by right atrium removal and heart perfusion with 5 mL of saline supplemented with 0.5 U/mL of heparin as previously described [13]. Organs were then excised, embedded in OCT compound (Tissue-Tek, Sakura Finetek, Torrance, CA, USA) and snap-frozen on dry ice.

### 2.9. Histological Analysis

To assess the level of fibrosis within the infarct zone, Masson’s trichrome stain was performed on 8 µm frozen sections of the heart. For that purpose, sections were washed with PBS and fixed for 1 h at RT in 10% buffered formalin solution and then incubated in Bouin’s solution (Sigma-Aldrich) for 30 min at 37 °C. All further steps were performed using the trichrome stain (Masson) kit (Sigma-Aldrich) according to the manufacturer’s protocol. The level of fibrosis was calculated based on histological analysis of heart sections. Each dot represents a single section from 2–4 animals. Scans of each stained section were collected using an LMD7000 Leica Microsystem microscope. The level of fibrosis was assessed using QuPath software [18] and calculated as the ratio of the area of blue-stained tissue to the entire surface area of the analyzed section. For ADSC-treated hearts, samples from 2 surviving animals were analyzed, whereas, for saline and hiPSC-CM-treated hearts, samples from 3–4 animals were assessed. In each group, from 3 to 23 sections were analyzed.

### 2.10. Immunohistochemical Analysis

To assess the presence of human cells within murine myocardium, the proliferation of injected cells and the number of alpha smooth muscle actin (αSMA)-positive vessels, the frozen 8 µm sections were washed in PBS, fixed in cold (−20 °C) acetone for 10 min and incubated for 30 min at RT in 0.2% Sudan Black B solution prepared as described previously [19]. Specimens were washed 5 times in water, blocked in Mouse on Mouse (MOM, Vector Laboratories, Burlingame, CA, USA) solution for 30 min at RT and then in 10% goat or donkey serum (Sigma-Aldrich) diluted in PBS for 1 h at RT and incubated overnight at 4 °C with primary antibodies 1:200 rabbit anti-human Ku80 (Abcam, Cambridge, UK, clone EPR3468), 1:200 rabbit anti-αSMA (Abcam, polyclonal), 1:100 FITC-conjugated mouse anti-Ki67 (BD Biosciences, San Jose, CA, USA, clone: B56), 1:100 anti-connexin 43 (Cx43, Abcam, polyclonal), 1:100 anti-α-actinin (Sigma-Aldrich, clone EA-53), anti-NKX2.5 (Santa Cruz Biotechnology, Dallas, TX, USA, polyclonal), 1:200 anti-GATA4 (Santa Cruz Biotechnology, polyclonal) and 1:200 mouse anti-cardiac troponin T (ThermoFisher Scientific, clone: 13-11) diluted in 1% goat or donkey serum. After washing 5 times with PBS, samples were incubated for 1 h at RT with secondary antibodies AlexaFluor568 goat anti-rabbit (for human Ku80 detection), AlexaFluor488 goat anti-rabbit (for αSMA detection), AlexaFluor568 goat anti-mouse, AlexaFluor647 donkey anti-mouse, AlexaFluor568 donkey anti-rabbit, AlexaFluor488 donkey anti-mouse (all from ThermoFisher Scientific) diluted in 1% goat or donkey serum with 0.2 µg/mL DAPI. Donkey serum and donkey-originated secondary antibodies were used for high-resolution images. Of note, AlexaFluor488-conjugated secondary antibody used as GFP in hiPSC-CMs was quenched after fixation of sections (data not shown). Sections were eventually mounted (DAKO Fluorescence mounting medium) and visualized under a fluorescent microscope (Nikon Eclipse TS100). The number of αSMA-positive vessels per mm^2^ of tissue was calculated using ImageJ software [20]. In each group (saline- and hiPSC-CM-treated hearts) samples from 2–3 animals were analyzed (5–12 sections per animal).

### 2.11. Statistical Analysis

Data are presented as mean ± SEM. To analyze statistical significance, a one-way ANOVA followed by Bonferroni’s post hoc test for multiple comparisons was used. Survival curves were analyzed by log-rank test. Statistical analyses were performed using GraphPad Prism software. *p* < 0.05 was considered as statistically significant.

## 3. Results

### 3.1. Generation of Genetically Modified ADSCs

The goal of this study was to verify the claimed, but not really proven, regenerative potential of adipose stromal cells in the injured heart and to determine whether the suggested therapeutic effect of hiPSC-CMs can be improved by overexpression of cardioprotective, proangiogenic and immunomodulatory factors, namely HO-1 and SDF-1α. Accordingly, in two sets of experiments, we determined whether overexpression of HO-1 and SDF-1α could influence the effect of human ADSC and hiPSC-CM administration in a murine model of acute MI. 

**Figure 1:**
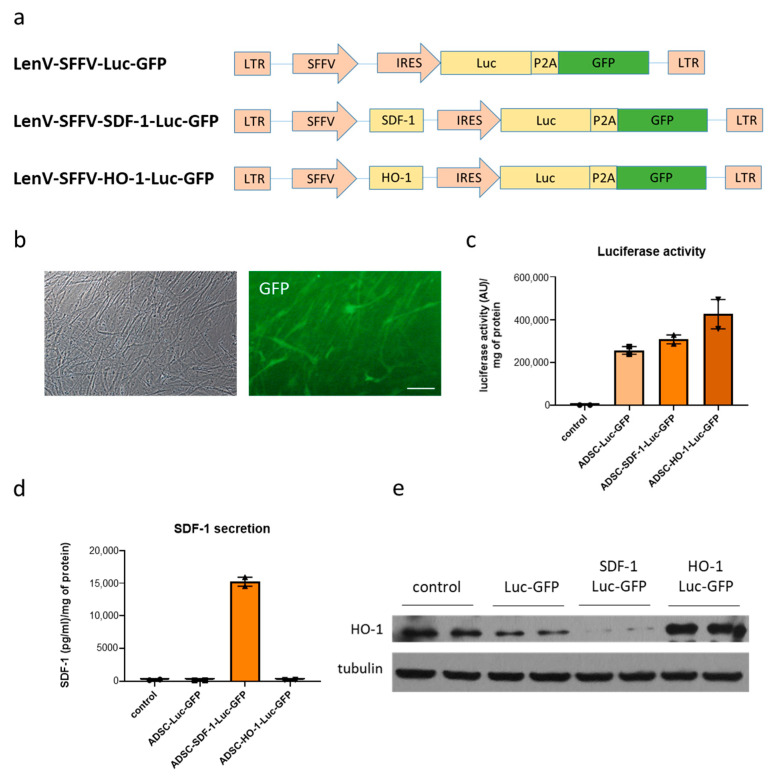
Generation of genetically modified adipose-derived stromal cells (ADSCs). (**a**) Schematic representation of lentiviral vectors used in the study. (**b**) GFP expression in ADSCs-Luc-GFP after cell sorting. Activity of luciferase (**c**) as well as expression of SDF-1 (**d**) and HO-1 (**e**) in transduced and purified ADSCs. Control—nontransduced ADSCs. Scale bar—50 µm.

In the first step, lentiviral vectors were constructed to allow simultaneous expression of a gene of interest, luciferase (enabling in vivo imaging) and GFP (for in vitro cell sorting). Particularly, Gibson Assembly method [21] was used to add luciferase-P2A fragment into LeGO-iG2 plasmid, and then standard molecular cloning allowed for the introduction of *HMOX1* and *CXCL12* coding sequences upstream of SFFV promoter (Figure 1a). In parallel, ADSCs were isolated from liposuction aspirate and cultured in a medium supplemented with 10% human platelet lysate. Importantly, this population demonstrated expression of common surface markers characteristic of mesenchymal cells (data not shown) as described by Czapla et al. [15]. Generated lentiviral vectors were used to transduce ADSCs, and GFP-expressing cells were then sorted (Figure 1b) and further expanded. Importantly, subsequent analyses of obtained genetically modified cell populations confirmed: luciferase activity in ADSCs-Luc-GFP, ADSCs-SDF-1-Luc-GFP and ADSCs-HO-1-Luc-GFP (Figure 1c); SDF-1α overexpression and secretion into the culture medium in ADSCs-SDF-1-Luc-GFP (Figure 1d); and HO-1 overexpression in ADSCs-HO-1-Luc-GFP (Figure 1e). These cells were therefore utilized in the next step to assess the therapeutic potential of ADSCs in the murine model of acute MI. Interestingly, a lower level of HO-1 was observed in SDF-1α-overexpressing cells (Figure 1e); however, further investigation of SDF-1α-HO-1 crosstalk was beyond the scope of this study.

### 3.2. Genetically Modified ADSCs Fail to Improve Heart Function in Nude Mice Subjected to MI

**Figure 2:**
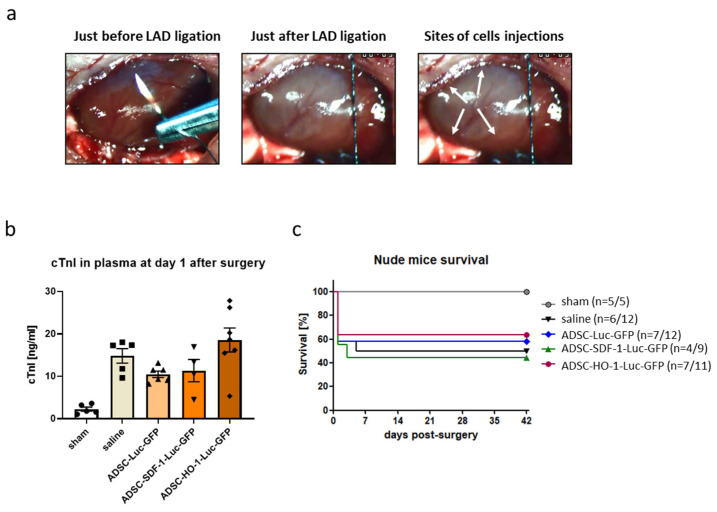
Induction of myocardial infarction (MI) in athymic *Nude* mice. (**a**) Schematic representation of murine heart subjected to MI. Arrows indicate the site of ADSC injections. (**b**) Level of cTnI in plasma of mice subjected to surgery and cell administration confirming successful MI induction. N = 4–7 animals/group. (**c**) Kaplan–Meier survival curves for *Nude* mice subjected to MI and cell administration. Sham-treated animals served as a control. N = 4–7 animals/group (numbers in parentheses represent surviving mice/all mice used).

In the first step, we assessed the potential of ADSCs to be retained in the murine heart. For that purpose, 5 × 10^5^ ADSCs-Luc-GFP were injected into control myocardium of athymic *Nude* immunocompromised mice followed by intraperitoneal administration of luciferin and intravital measurement of bioluminescence 72 h after the procedure. No luciferase activity was observed, indicating that ADSCs fail to be retained in the murine hearts (Supplementary Figure S1). Nevertheless, as some reports described a beneficial effect of mesenchymal stromal cell administration regardless of their limited survival [22] and in order to assess the effect of SDF-1α and HO-1 overexpression on the therapeutic potential of ADSCs, we proceeded further with in vivo experiments. In the first set, MI was induced in *Nude* mouse strain. Particularly, after exposing the heart, LAD was permanently ligated with a single silk suture (Figure 2a, left panel). This resulted in an instant color change of the myocardial area below the ligation site from bright red to white, indicating blockage of blood supply (Figure 2a, middle panel) and providing borders of the infarct zone for cell delivery (Figure 2a, right panel). Genetically modified ADSCs, suspended in saline, were injected in four sites (5 × 10^5^ cells in total) immediately after LAD ligation. After 24 h, blood was collected from animals that underwent surgery, and the level of troponin I in the plasma was measured, confirming the successful induction of MI (Figure 2b). Mice were further monitored for 6 weeks; however, no significant differences in survival were observed between control animals subjected to MI and saline injection and the groups treated with genetically modified ADSCs, regardless of therapeutic transgene overexpressed in the cells (Figure 2c). Of note, the sharp decrease in survival rate in the first hours/days after MI was likely associated with the surgery itself and susceptibility of *Nude* mice to MI-driven mortality in our experimental model. It might have been also related to poor 2,2,2-tribromoethanol tolerance in this strain of mice subjected to MI (no mortality was observed in sham-operated animals).

**Figure 3:**
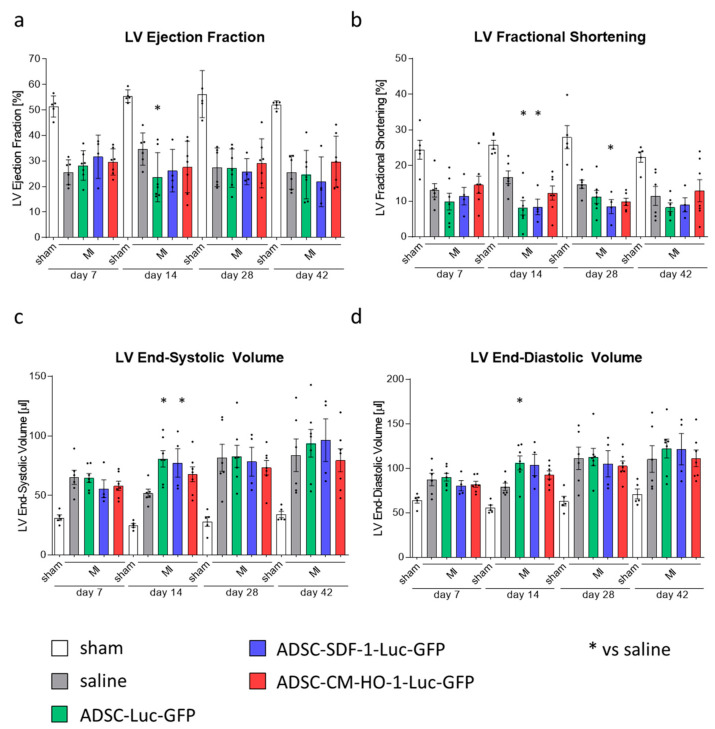
No improvement of heart function after administration of genetically modified ADSCs. Parameters of left ventricle (LV) function: ejection fraction (**a**), fractional shortening (**b**), end-systolic volume (**c**) and end-diastolic volume (**d**) in animals subjected to MI and administration of cells. N = 4–7 animals/group. * *p* < 0.05 vs. saline, one-way ANOVA with Bonferroni’s post hoc test.

To assess the effect of cell therapy on the heart function, transthoracic echocardiography was performed on days 7, 14, 28 and 42 after MI. The analyses revealed a substantial decrease of left ventricle (LV) ejection fraction (LVEF) and LV fractional shortening (LVFS) as well as an increase in LV end-systolic volume (LVVs) and LV end-diastolic volume (LVVd) in all mice subjected to MI, at all tested time-points, confirming the deterioration of heart function in these animals (Figure 3a–d; Supplementary Figure S2). None of these parameters, however, improved after administration of genetically modified ADSCs, neither control (ADSCs-Luc-GFP) nor those overexpressing HO-1 or SDF-1α (ADSCs-HO-1-Luc-GFP and ADSCs-SDF-1-Luc-GFP, respectively) at any of the time-points analyzed (Figure 3a–d). Interestingly, some of the parameters deteriorated even further on day 14 after MI induction in mice treated with ADSCs-Luc-GFP (LVEF, LVFS, LVVs and LVVd; Figure 3a–d) and ADSCs-SDF-1-Luc-GFP (LVFS and LVVs; Figure 3b,c). Obtained results showed that ADSCs do not have any therapeutic potential in this acute model of MI and neither HO-1 nor SDF-1α overexpression provides a beneficial effect in these cells.

### 3.3. Generation of Genetically Modified hiPSCs

**Figure 4:**
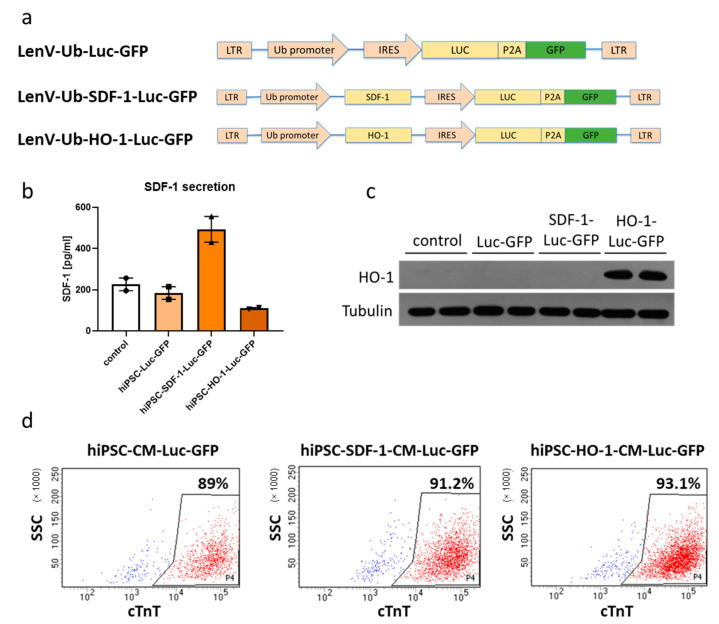

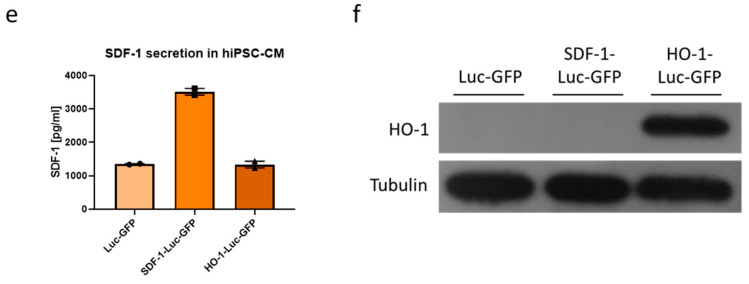
Generation of genetically modified human induced pluripotent stem cell-derived cardiomyocytes (hiPSC-CMs). (**a**) Schematic representation of lentiviral vectors used to transduce hiPSCs. Secretion of SDF-1α (**b**) and expression of HO-1 (**c**) in transduced hiPSCs. (**d**) Cardiac differentiation efficiency of transduced and sorted hiPSCs measured as percentage of cells positive for cardiac troponin T (cTnT). Secretion of SDF-1α (**e**) and expression of HO-1 (**f**) in genetically modified hiPSC-CMs.

ADSCs, regardless of transgene overexpression, did not provide therapeutic activity in the MI model used in this study. These cells, however, do not demonstrate the capacity to differentiate into cardiomyocytes, which appears to substantially limit their potential to regenerate infarcted myocardium [4]. Thus, we decided to additionally assess the therapeutic effect of genetically modified hiPSC-CMs in a similar experimental setting. For that purpose, hiPSCs were reprogrammed from PBMCs of a healthy donor using non-integrating Sendai vectors. Importantly, generated cells expressed pluripotency markers (OCT4, NANOG, SSAE4, TRA-1-60, TRA-1-81 and alkaline phosphatase; Supplementary Figure S3a) and spontaneously differentiated in vitro via embryoid bodies into cells originating from three germ layers (Supplementary Figure S3b). Thus, in the next step, hiPSCs were transduced with the same lentiviral vectors applied to overexpress HO-1 or SDF-1α together with luciferase and GFP in ADSCs. Transgene expression, however, was readily silenced in hiPSCs (data not shown). Thus, the Luc-GFP, HO-1-Luc-GFP and SDF-1α-Luc-GFP polycistronic cassettes were transferred into FUW (flap-Ub promoter-WRE) plasmid backbone, upstream of ubiquitin C promoter (Figure 4a), and further used to produce lentiviral vectors. After transduction of hiPSCs, cells were sorted based on GFP expression and expanded. Upon confirmation of SDF-1α (Figure 4b) and HO-1 (Figure 4c) overexpression, genetically modified hiPSCs were subjected to cardiac differentiation using small molecules regulating the WNT pathway. The process was highly efficient as approximately 90% of cardiac troponin T-positive cells were obtained from each modified hiPSC line (Figure 4d). Using hiPSC-CMs generated from parental hiPSC line, we confirmed the reliability of flow-cytometric-based assessment of differentiation efficiency, as high yield of cardiac troponin T-positive cells (Supplementary Figure S4a) was paralleled with the homogenous expression of cardiac markers, including NKX2.5, α-actinin and cardiac troponin T (Supplementary Figure S4b). At this stage of development, obtained cardiomyocytes demonstrated an embryonic-like phenotype; however, high-resolution imaging revealed the presence of well-organized sarcomeric structures (Supplementary Figure S4c). Importantly, further analyses of genetically modified hiPSC-CMs confirmed SDF-1α overexpression and its secretion into the culture medium in hiPSC-CMs-SDF-1-Luc-GFP (Figure 4e) and HO-1 overexpression in hiPSC-CMs-HO-1-Luc-GFP (Figure 4f). Due to the application of different promoters in lentiviral vectors used to transduce ADSCs (SFFV-derived) and hiPSCs (ubiquitin C), the levels of transgenes were not compared in genetically modified ADSCs and hiPSC-CMs. On the other hand, these cells were utilized in the next step to assess their therapeutic potential in the murine model of acute MI, also including an ADSC-Luc-GFP population for a direct comparison between hiPSC-CMs and ADSCs.

### 3.4. Genetically Modified hiPSC-CMs Improve Heart Function in NOD/SCID Mice Subjected to MI

**Figure 5:**
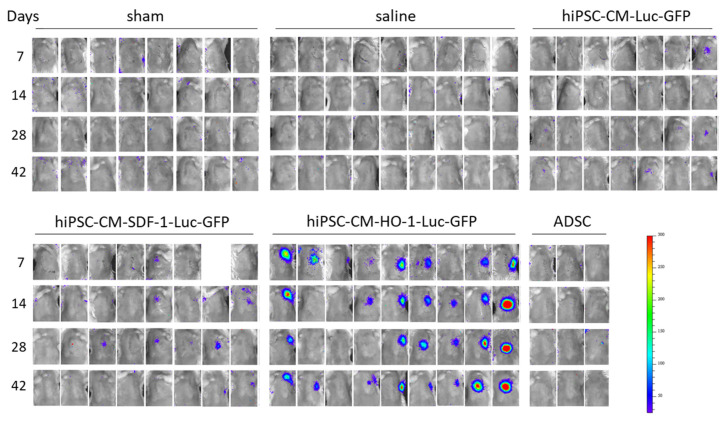
Detection of human cells in murine hearts in vivo. Bioluminescent signal collected intravitally on days 7, 14, 28 and 42 after MI induction from the chest of all animals used in the experiment. N = 3 (ADSC) or 7–9 animals/group.

**Figure 6:**
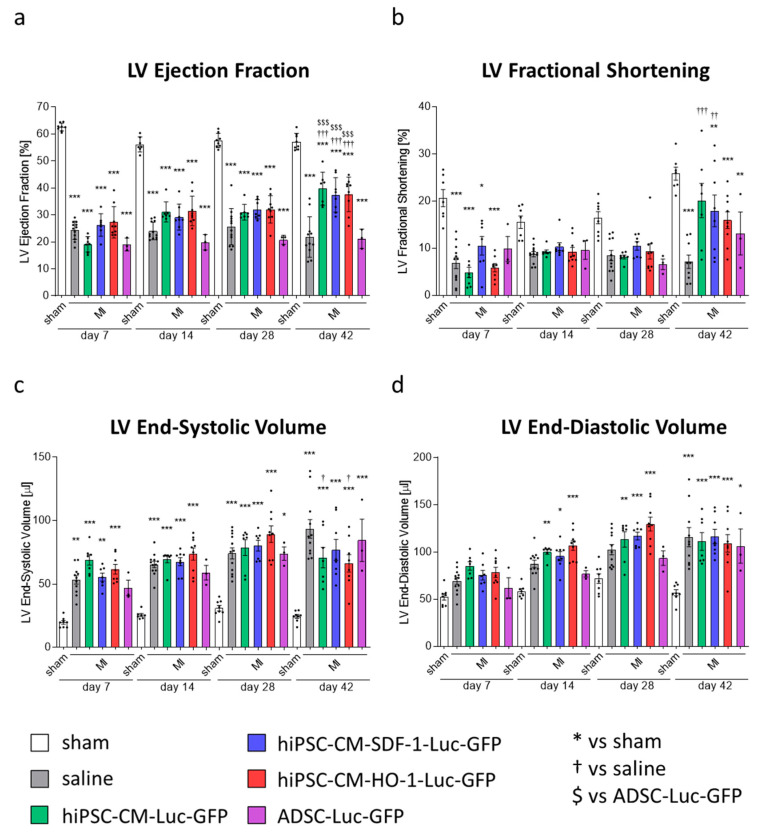
Administration of hiPSC-CMs improves heart function 42 days after MI. (**a**) LV ejection fraction (LVEF) measured 7, 14, 28 and 42 days after MI induction and administration of genetically modified hiPSC-CMs and ADSCs. N = 3 (ADSC) or 7–12 animals/group. *** *p* < 0.005 vs. sham group; ^†††^ *p* < 0.005 vs. saline group; ^$$$^ *p* < 0.005 vs. ADSC-Luc-GFP group; one-way ANOVA with Bonferroni’s post hoc test. (**b**) LV fractional shortening (LVFS) measured 7, 14, 28 and 42 days after MI induction and administration of genetically modified hiPSC-CMs and ADSCs. N = 3 (ADSC) or 7–12 animals/group. * *p* < 0.05, ** *p* < 0.01, *** *p* < 0.005 vs. sham group; ^††^ *p* < 0.01, ^†††^ *p* < 0.005 vs. saline group; one-way ANOVA with Bonferroni’s post hoc test. (**c**) LV end-systolic volume (LVVs) measured 7, 14, 28 and 42 days after MI induction and administration of genetically modified hiPSC-CMs and ADSCs. N = 3 (ADSC) or 7–12 animals/group. * *p* < 0.05, ** *p* < 0.01, *** *p* < 0.005 vs. sham group; ^†^ *p* < 0.05 vs. saline group; one-way ANOVA with Bonferroni’s post hoc test. (**d**) LV end-diastolic volume (LVVd) measured 7, 14, 28 and 42 days after MI induction and administration of genetically modified hiPSC-CMs and ADSCs. N = 3 (ADSC) or 7–12 animals/group. * *p* < 0.05, ** *p* < 0.01, *** *p* < 0.005 vs. sham group; one-way ANOVA with Bonferroni’s post hoc test.

As previous analysis indicated no ADSC retention in hearts of *Nude* mice, we used the *NOD/SCID* immunocompromised murine strain in the second set of in vivo experiments. *NOD/SCID* mice, in contrast to athymic *Nude* animals, lack not only T-cells but also B-cells and show decreased NK cell activity; thus, they are more receptive for human cell xenografts [23]. MI induction and cell administration were performed as described for the first set of in vivo experiments (Figure 2a); however, here we tested the therapeutic effect of genetically modified hiPSC-CMs and ADSCs-Luc-GFP. ADSCs-SDF-1-Luc-GFP and ADSCs-HO-1-Luc-GFP were not included, as overexpression of SDF-1α and HO-1 did not provide improvement in heart function upon cell administration in the previous experiment. In general, we observed a better post-MI survival of NOD/SCID (60–80%; Supplementary Figure S5) than of *Nude* (approx. 40–60%; Figure 2C) mice. The lowest survival (high mortality especially in the first hours after the surgery) was observed in ADSC-Luc-GFP-treated NOD/SCID mice (Supplementary Figure S5). At the end of the experiment, however, differences between MI-subjected groups were not statistically significant. To assess the presence of injected cells, measurement of luciferase activity was additionally included alongside transthoracic echocardiography. This analysis performed on days 7, 14, 28 and 42 post-MI revealed persistent bioluminescence signal detected at all tested time points in five out of nine mice injected with hiPSC-CMs-HO-1-Luc-GFP, while the signal was observed on selected days in other animals treated with hiPSC-CMs (Figure 5). No signal was detected in mice receiving either saline (as expected) or ADSCs-Luc-GFP throughout the measurements (Figure 5). Importantly, direct evaluation of luciferase activity in hearts explanted 42 days after MI induction confirmed the presence of hiPSC-CMs-Luc-GFP, hiPSC-CMs-HO-1-Luc-GFP and hiPSC-CMs-SDF-1-Luc-GFP in the murine myocardium for at least 6 weeks after administration, and the highest mean signal was observed in hiPSC-CM-HO-1-Luc-GFP-treated hearts (Supplementary Figure S6a,b). Echocardiographic analyses (Supplementary Figure S7) performed on days 7, 14, 28 and 42 after MI induction and cell administration revealed substantial improvement of LVEF in mice injected with hiPSC-CMs-Luc-GFP, hiPSC-CMs-SDF-1-Luc-GFP and hiPSC-CMs-HO-1-Luc-GFP occurring between days 28 and 42 post-MI (Figure 6a). Similarly, LVFS was improved after administration of control and SDF-1α-overexpressing hiPSC-CMs (Figure 6b), whereas LVVs was improved after administration of control and HO-1-overexpressing cells (Figure 6c) at day 42. No significant changes in LVVd were observed between all MI groups (Figure 6d), indicating similar LV dilation in all animals 42 days after MI. No beneficial effect in any of the assessed echocardiographic parameters was detected in animals treated with ADSCs-Luc-GFP (Figure 6a–d). In summary, echocardiography analyses revealed significant improvement of contractile capacity (LVEF) at day 42 post-MI in hiPSC-CM-treated animals, but not in ADSC-treated ones. Of note, application of hiPSC-CMs provided the therapeutic outcome only 4 weeks after MI induction (between days 28 and 42), while at earlier time points the heart function of the hiPSC-CM-treated mice was similar to that of the group subjected to MI and saline injection.

**Figure 7:**
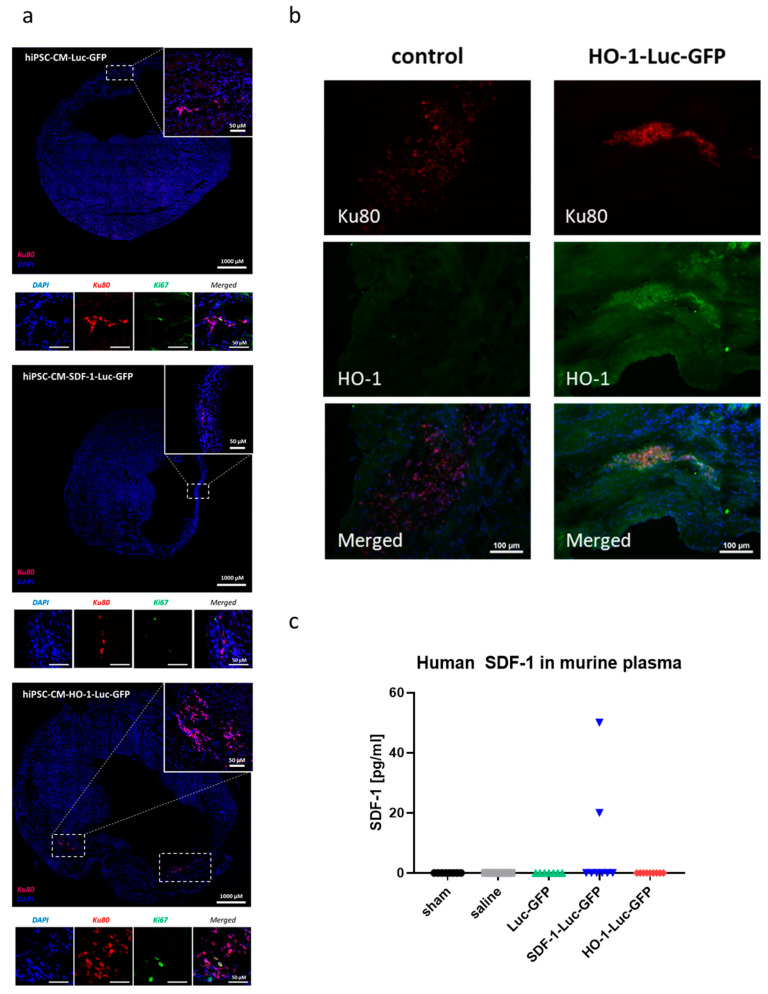
Immunofluorescent analysis of hearts subjected to MI and cell therapy. (**a**) Immunofluorescent analysis of human Ku80 (red) as well as Ki67 (green) in murine hearts subjected to MI and injected with hiPSC-CMs-Luc-GFP, hiPSC-CMs-SDF-1-Luc-GFP and hiPSC-CMs-HO-1-Luc-GFP. Nuclei stained with DAPI (blue). (**b**) Immunofluorescent analysis of Ku80 (red) and HO-1 expression (green) in hearts treated with either control hiPSC-CMs or hiPSC-CMs-HO-1-Luc-GFP. Nuclei stained with DAPI (blue). (**c**) Human SDF-1α-targeted ELISA assay performed on plasma collected from sham as well as saline-, hiPSC-CM-GFP-Luc-GFP-, hiPSC-CM-SDF-1-Luc-GFP- and hiPSC-CM-HO-1-Luc-GFP-treated animals 42 days after MI induction. N = 7–9 animals/group.

**Figure 8:**
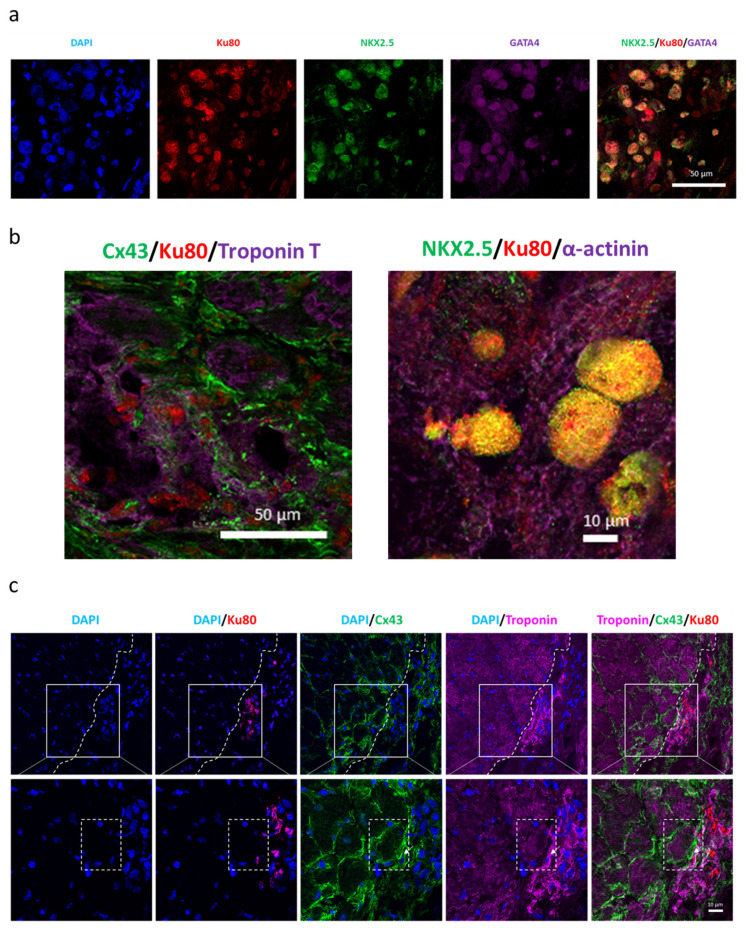
Expression of cardiac markers in human cells detected in murine myocardium. (**a**) Representative pictures for immunofluorescent analysis of human Ku80 (red), NKX2.5 (green) and GATA4 (purple) in murine heart subjected to MI and hiPSC-CM-SDF-1-Luc-GFP administration. Nuclei stained with DAPI (blue). (**b**) Representative pictures for immunofluorescent analysis of human Ku80 (red), connexin 43 (Cx43, green) and troponin T (purple) in the left panel and NKX2.5 (green) and α-actinin (purple) in the right panel in murine heart subjected to MI and hiPSC-CM-SDF-1-Luc-GFP administration. Nuclei stained with DAPI (blue). (**c**) Representative pictures for immunofluorescent analysis of human Ku80 (red), connexin 43 (Cx43, green) and troponin T (purple) in murine heart subjected to MI and hiPSC-CM-SDF-1-Luc-GFP administration. Nuclei stained with DAPI (blue). Similar results were obtained for hiPSC-CMs-Luc-GFP and hiPSC-CMs-HO-1-Luc-GFP (Supplementary Figure S9).

Immunofluorescent analysis with human-specific anti-Ku80 antibody performed on frozen sections additionally confirmed the presence of genetically modified hiPSC-CMs within murine myocardium, most probably at the sites of injection (Figure 7a). Interestingly, single cells in each group were also positive for proliferation marker Ki67 (Figure 7a), suggesting the proliferation of injected hiPSC-CMs. Of note, immunofluorescent analysis of HO-1 level confirmed overexpression of this protein in hiPSC-CMs-HO-1-Luc-GFP injected in the murine heart (Figure 7b). Similarly, human SDF-1α-targeted ELISA assay performed on plasma samples collected on day 42 after MI induction revealed the presence of this chemokine in two mice treated with hiPSC-CMs-SDF-1-Luc-GFP (Figure 7c). Visualization of human cells in murine tissue indicated also that control and HO-1- and SDF-1-overexpressing hiPSC-CMs were localized in close proximity to the host myocardium (Supplementary Figure S8), suggesting possible direct interactions between human and mouse cardiomyocytes. Importantly, high-resolution imaging revealed the presence of cardiomyocyte markers, including NKX2.5 and GATA4 (Figure 8a) as well as troponin T and α-actinin, organized in sarcomeric structures, (Figure 8b) in Ku80-expressing grafted cells (Supplementary Figure S9). Connexin 43 was also detected in hiPSC-CMs within murine myocardium with direct interactions between human cells and murine cardiomyocytes (Figure 8c, Supplementary Figure S9).

**Figure 9:**
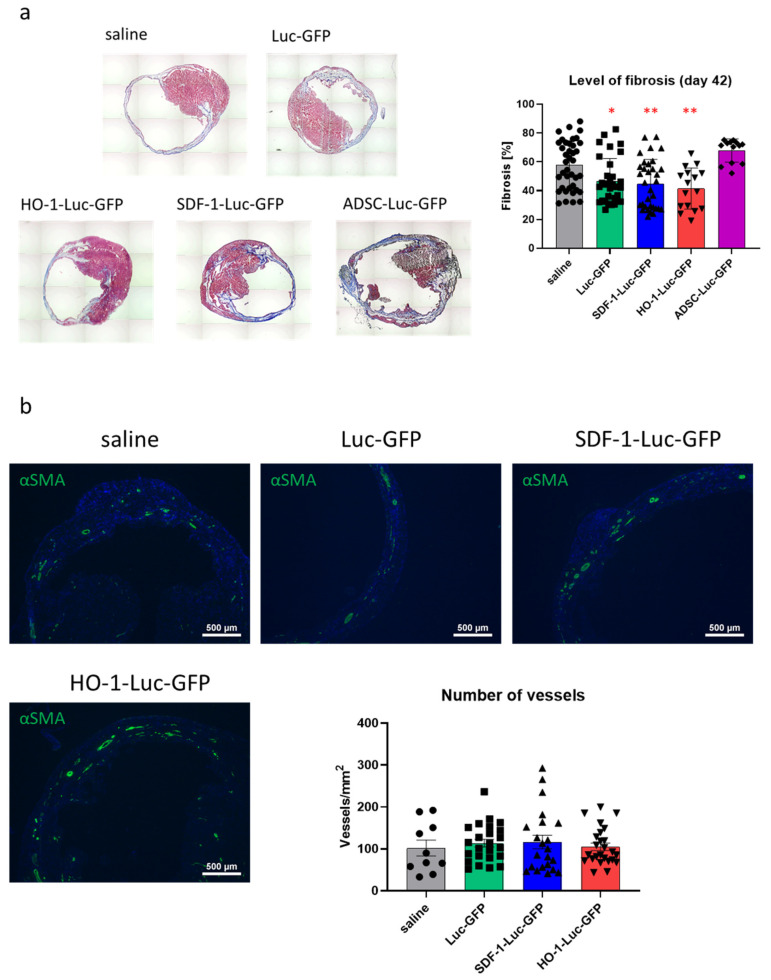
Histological analysis of hearts subjected to MI and cell therapy. (**a**) Masson’s trichrome analysis of heart sections obtained from animals subjected to MI and cell therapy—representative pictures. Level of fibrosis was calculated based on histological analysis of heart sections. N = 2 (ADSC) or 4 animals/group, each dot represents a single section, 3–23 sections/heart were analyzed. * *p* < 0.05, ** *p* < 0.01 vs. saline. (**b**) Representative pictures for immunofluorescent analysis of αSMA-positive vessels (green) in left ventricle of saline-, hiPSC-CM-GFP-Luc-GFP-, hiPSC-CM-SDF-1-Luc-GFP- and hiPSC-CM-HO-1-Luc-GFP-treated hearts. Nuclei stained with Hoechst33342 (blue). Data presented as number of vessels/mm^2^. N = 2–3 animals/group, each dot represents a single section, 5–12 sections/animal were analyzed.

Histological analysis with Masson’s trichrome staining further revealed a decreased level of fibrosis indicating reduced infarct size in hearts treated with hiPSC-CMs-Luc-GFP, hiPSC-CMs-SDF-1-Luc-GFP and hiPSC-CMs-HO-1-Luc-GFP when compared with the saline-injected group (Figure 9a), corroborating echocardiographic measurements. Infarct size in ADSC-Luc-GFP-treated hearts remained unchanged (Figure 9a), again undermining the claimed therapeutic effect of ADSCs in MI. On the other hand, the number of vessels in hiPSC-CM-treated mice and saline-injected mice was similar (Figure 9b).

## 4. Discussion

The salient finding of the present study is the demonstration that hiPSC-CMs can ameliorate the development of heart failure after myocardial infarction while the ADSCs are ineffective. The work adds to elucidating the potential of different cell types tested for the treatment of heart injury. 

Numerous studies have focused so far on the utilization of so-called mesenchymal stromal cells, improperly named generally as “stem cells”; however, due to their inability to differentiate into cardiomyocytes and limited survival upon in vivo administration, no effective treatment targeting myocardial infarction has been developed so far [4]. Importantly, the results from the current study further corroborate these findings, as neither native ADSCs nor ADSCs overexpressing cardioprotective, proangiogenic and immunomodulatory factors provided improved heart function in the model of acute MI. A similar lack of therapeutic potential in two immunocompromised murine strains, namely *Nude* and *NOD/SCID*, indicates that the reported failure to provide beneficial effects did not depend on the receptiveness of mice to human cells.

In contrast to ADSCs, cardiomyocytes generated from pluripotent stem cells represent biologically justified candidates for cell therapy, as these cells are lost and do not regenerate after heart injury. Previous studies have also demonstrated that genetic or electromechanical conditioning of human embryonic stem cell (hESC)- or hiPSC-derived cardiomyocytes enhances their therapeutic potential in animal MI models. For instance, genetically modified hiPSC-CMs expressing N-cadherin enhanced LVEF and decreased infarct size more profoundly than nonmodified cells [24].

In this study, we hypothesized that overexpression of HO-1 and SDF-1α would further enhance the regenerative potential of hiPSC-CMs. The rationale for overexpression of *HMOX-1*, encoding HO-1, was based on our previous studies demonstrating that lack of HO-1 resulted in adverse late LV remodeling due to overactive and prolonged postischemic inflammatory response [13]. Reversely, when Yet et al. subjected transgenic mice with cardiac-specific overexpression of human HO-1 to ischemic/reperfusion injury of the heart, the improved contractile activity in the reperfusion phase and reduced infarct size in these animals in comparison to the control counterparts was observed [25]. Of note, this phenotypic effect was underlain by decreased inflammatory cell infiltration and oxidative damage. Other reports also revealed an important role of HO-1 in the regulation of cardiomyocyte metabolism [12,26]. On the other hand, SDF-1α has been also described as potent cardioprotective chemokine acting mainly through binding to its cognate receptor CXCR4 and downstream activation of STAT3 transcription factor in the ischemia/reperfusion conditions [14].

Nonetheless, we did not observe that HO-1 or SDF-1α overexpression additionally augments the already substantial therapeutic potential of control hiPSC-CMs in the experimental settings used in the study. Interestingly, the most profound restoration of cardiac function in all groups receiving hiPSC-CMs was detected between day 28 and day 42 post-MI, highlighting the need for long-term evaluation of cell therapy effects. This appears to be an important observation as many studies terminate cardiac function measurements on day 28 after MI. Van Laake et al. raised a similar issue after describing that the therapeutic effect of hESC-CM administration in a murine model of acute MI is transient and can be observed 4 weeks after injection but not after 3 months [27]. Nevertheless, the improvement in cardiac function described in that study was much lower (LVEF in hESC-CM-treated animals was less than 30% on week 4) than in our experiments. Longer follow-up of our analysis may be essential to assess whether overexpression of either HO-1 or SDF-1α in hiPSC-CMs further augments LVEF in time points later than 6 weeks post-MI. To this end, however, we cannot exclude that the level of HO-1 and SDF-1α locally in murine myocardium was too low to further enhance heart function and that, within the time period tested, both factors do not provide a significant therapeutic effect in the applied experimental model.

Including longer time-points is also particularly important, as vector-based genetic modifications provide permanent expression of transgenes and thus generate a risk of detrimental impact of delivered factors on the administered cell population and/or surrounding tissue. Gabisonia et al., for instance, reported that AAV-based expression of human microRNA-199a (miR-199a) in pig myocardium subjected to MI resulted initially in significant improvement of heart function and decreased infarct size up to week 7 post-surgery, when sudden death of most of the animals occurred [28]. As miR-199a induces proliferation of cardiomyocytes, the initial therapeutic effect depended on the restoration of the proliferative capacity of swine nondamaged myocardium. Further histological analyses revealed that the same mechanism accounted for the formation of dividing cells, which displayed poorly differentiated myoblastic phenotype in the long term [28]. Similarly, in our previous study, we demonstrated that high persistent overexpression of HO-1 in murine myoblasts augmented their proliferation and upon in vivo administration led to the development of fast-growing, hyperplastic tumors infiltrating the surrounding tissues [29]. However, in the current study, we noticed only single Ki67-positive hiPSC-CMs that survived in murine myocardium up to day 42 post-MI, with no signs of tumor growth in any of the tested groups. Interestingly, we detected the highest signal of bioluminescence originating from luciferase expression in mice treated with HO-1-overexpressing hiPSC-CMs, which may indicate better survival of these cells in murine myocardium. This observation goes in line with those of Luo et al., who demonstrated that pretreatment of hESC-CMs with cobalt protoporphyrin IX (CoPPIX), a potent inducer of HO-1 expression, resulted in significantly larger graft size upon intramyocardial administration in the rat model of acute myocardial infarction in comparison to PBS-pretreated cells [30]. Interestingly, in another report, Kirby et al. searched for small molecules that can protect hiPSC-CMs from peroxide-induced cell death. Evaluation of 48,640 compounds revealed that the hit molecule strongly upregulated HO-1 expression [31], indicating that the regulation of HO-1 level in cardiomyocytes may increase their survival in oxidative stress conditions. Nevertheless, long-term studies in larger animal models are necessary to demonstrate the sustained beneficial effect and to exclude any detrimental influence of persistent HO-1 overexpression.

Vagnozzi et al. recently documented that the beneficial effect of cell therapy using fractionated bone marrow mononuclear cells (often used in human clinical trials) and cardiac mesenchymal cells in a murine model of ischemia/reperfusion heart injury does not depend on the delivered cells, which were cleared from the host tissue within two weeks after administration [32]. Particularly, the same effect was achieved with zymosan, a potent inducer of innate immune response, and with freeze/thawed killed cells, whereas further analyses confirmed that the improvement of cardiac function was dependent on the acute inflammation-based wound healing. However, our histological examinations revealed the presence of human cardiomyocytes in the murine hearts treated with control and either HO-1- or SDF-1α-overexpressing hiPSC-CMs. This strongly suggests that the beneficial effect of hiPSC-CM administration observed in our study was related to the activity of these cells, either paracrine and/or via direct effect on murine myocardium. Additionally, as we used immunocompromised animals, we may anticipate that the acute immune response was blunted and could not play such an important role. This would also explain the lack of the therapeutic effect upon injection of ADSCs, which were not detected in the murine myocardium based on the luciferase activity at any tested time points. We cannot, however, completely exclude the effect of macrophages at the site of injury and cell injection, as our study did not address the evaluation of the early innate immune response. Thus, further studies are needed to address the role of immune cells in hiPSC-CM- and ADSC-based cell therapy outcomes.

Detection of hiPSC-CMs in close proximity to murine myocardium surviving in the left ventricle after MI described in this study emphasizes possible direct interactions between human and mouse cardiomyocytes and thus a risk of pro-arrhythmogenic activity of delivered cells. Indeed, immunohistochemical analysis revealed the expression of connexin 43 in engrafted hiPSC-CMs with direct coupling of human cells with murine myocardium. An arrhythmogenic effect was already described for hESC-derived CMs administrated in large animal models, including pigs [33] and macaques [34], and therefore telemetric monitoring of heart rate would be of high importance for the future studies to comprehensively assess the effect of hiPSC-CMs and their genetic modifications on the electromechanical activity of host myocardium.

## 5. Conclusions

In summary, we demonstrated that hiPSC-CMs provide a substantial improvement in cardiac function in a murine model of acute MI. The most profound effect was observed between days 28 and 42 after MI induction, highlighting the need for long-term evaluation of any cell-based therapy effects on regenerating myocardium. Additionally, we did not detect any further beneficial outcome in animals treated with HO-1 and SDF-1α-overexpressing cells up to 6 weeks after administration. Nonetheless, further analyses are needed to determine the effect of these factors at later time points, especially because the strongest luminescence signal of hiPSC-CMs overexpressing HO-1 suggests their better functionality. Contrary to hiPSC-CMs, the delivery of ADSCs did not result in any improvement of heart function, putting into question further use of such cells in cardiac cell therapies.

## Supplementary Materials

The following are available online at https://www.mdpi.com/2227-9059/8/12/578/s1.
