# Supplementary material for: Human Induced Pluripotent Stem Cell-Derived Cardiomyocytes, in Contrast to Adipose Tissue-Derived Stromal Cells, Efficiently Improve Heart Function in Murine Model of Myocardial Infarction
Source: Biomedicines. 2020 Dec 7;8(12):578. doi: 10.3390/biomedicines8120578 (PMC7762393; doi:10.3390/biomedicines8120578)

Supplementary Material

1. **Supplementary Figure legends**

Supplementary Figure 1. Bioluminescent signal collected intravitally from control (N=2) and ADSC-Luc-GFP-injected (N=3) *Nude* mice 3 days after cell administration. No bioluminescence was detected.

Supplementary Figure 2. Representative echocardiography of left ventricles at systolic and diastolic phase of *Nude* mice exposed to sham or MI surgery receiving control solution (PBS) or ADSC-Luc-GFP, ADSC-SDF-1-Luc-GFP, ADSC-HO-1-Luc-GFP. Analysis performed 42 days after the surgery and intracardiac cells injection. Green and red lines indicate left ventricular lumens at systolic and diastolic phase, respectively.

Supplementary Figure 3. (a) Immunofluorescent analysis of pluripotency markers: OCT4 (red, upper left), NANOG (green, upper right), SSEA4 (green, middle left), TRA-1-60 (green, middle right), TRA-1-81 (green, bottom left) and analysis of alkaline phosphatase activity (ALP, red, bottom right) in hiPSC used in the study. Nuclei stained with Hoechst33342 (blue). Scale bar – 50 µm. (b) Immunofluorescent analysis of GATA binding protein 4 (GATA4, red, mesodermal and endodermal marker), Neurofilament heavy polypeptide (NFH, green, ectodermal marker) and Alpha-fetoprotein (AFP, green, endodermal marker) expression in spontaneously differentiating hiPSC. Nuclei stained with Hoechst33342 (blue). Scale bar – 50 µm.

Supplementary Figure 4. (a) Flow cytometric-based analysis of cardiac differentiation efficiency of parental hiPSC line measured as percentage of cells positive for cardiac troponin T (cTnT). (b) Representative pictures for immunofluorescent analysis of α-actinin (green, left panel), troponin T (green, right panel) and NKX2.5 (red) in hiPSC-CM generated from parental hiPSC-CM. The same cells were used in parallel for flow cytometric-based assessment of cardiac differentiation efficiency. Nuclei stained with DAPI (blue). (c) High resolution imaging of hiPSC-CM generated from parental hiPSC line: of α-actinin (green, upper panel), troponin T (green, bottom panel) and NKX2.5 (red). Nuclei stained with DAPI (blue).

Supplementary Figure 5. Kaplan-Meier survival curves for *NOD/SCID* mice subjected to MI and administration of saline, hiPSC-CM-Luc-GFP, hiPSC-CM-SDF-1-Luc-GFP, hiPSC-CM-HO-1-Luc-GFP or ADSC-Luc-GFP. Sham mice served as a control. N=7–14 animals/group (numbers in parentheses – surviving mice/all mice used).

Supplementary Figure 6. (a) Bioluminescent signal collected from hearts explanted 42 days after MI induction and administration of hiPSC-CM-Luc-GFP, hiPSC-CM-SDF-1-Luc-GFP, hiPSC-CM-HO-1-Luc-GFP or ADSC-Luc-GFP. Sham and saline-injected animals served as a control. (b) Data presented as total counts of bioluminescence detected within 10 min of measurements. N=2 (ADSC) or 7–9 hearts/group.

Supplementary Figure 7. Representative echocardiography of left ventricles at systolic and diastolic phase of *NOD/SCID* mice exposed to sham or MI surgery receiving control solution (saline) or hiPSC-CM-Luc-GFP; hiPSC-CM-SDF-1-Luc-GFP, hiPSC-CM-HO-1-Luc-GFP or ADSC-Luc-GFP. Analysis performed 42 days after the surgery and intracardiac cells injection. Green and red lines indicate left ventricular lumens at systolic and diastolic phase, respectively.

Supplementary Figure 8. Representative pictures for immunofluorescent analysis of human Ku80 (green in hiPSC-CM-Luc-GFP-treated heart and red for hiPSC-CM-SDF-1-Luc-GFP- and hiPSC-CM-HO-1-Luc-GFP-treated heart) and cardiac troponin T (red in hiPSC-CM-Luc-GFP-treated heart and green for hiPSC-CM-SDF-1-Luc-GFP- and hiPSC-CM-HO-1-Luc-GFP-treated heart) in murine heart subjected to MI and hiPSC-CM-Luc-GFP, hiPSC-CM-SDF-1-Luc-GFP and hiPSC-CM-HO-1-Luc-GFP administration. Nuclei stained with DAPI (blue). N=7–9 animals/group.

Supplementary Figure 9. Representative pictures for immunofluorescent analysis of human Ku80 (red), NKX2.5 (green) and α-actinin (purple); human Ku80 (red), NKX2.5 (green) and troponin T (purple) as well as human Ku80 (red), connexin 43 (Cx43, green) and troponin T (purple) in murine hearts subjected to MI and either hiPSC-CM-Luc-GFP (upper panel) or hiPSC-CM-HO-1-Luc-GFP (bottom panel) administration. Nuclei stained with DAPI (blue).

2. Supplementary Methods

2.1. Construction of plasmids for lentiviral vectors production

To transduce ADSC with lentiviral vectors in the first step the pLeGO-iLG2 plasmid was constructed using Gibson Assembly method. For that purpose i) coding sequence of luciferase was amplified from pLenti-Luc plasmid (kindly gifted by Department of Transplantation; Intitute of Pediatrics, Jagiellonian University Medical College, Krakow, Poland) using primers attaching P2A sequence to its 3' end (forward: 5' CCA CAA CCG CCA CCA TGG AAG ACG CCA AAA ACA TAA AG 3'; reverse: 5' AGG TCC AGG GTT CTC CTC CAC GTC TCC AGC CTG CTT CAG CAG GCT GAA GTT AGT AGC TCC GCT TCC CAC GGC GAT CTT TCC GCC CTT C 3'), ii) GFP coding sequence was amplified from LeGO-iG2 plasmid (Addgene #27341 [1]) using primers attaching P2A sequence to its 5' end (forward: 5' GGA AGC GGA GCT ACT AAC TTC AGC CTG CTG AAG CAG GCT GGA GAC GTG GAG GAG AAC CCT GGA CCT ATG GTG AGC AAG GGC GAG GAG C 3'; reverse: 5' TTA CTT GTA CAG CTC GTC CAT GC 3'), iv) both fragments were isolated from the agarose gel, combined and Luc-P2A-GFP sequence was amplified using luciferase primer forward and GFP primer reverse (overlap PCR) and iv) LeGO-iG2 backbone using primers designed to contain proper overlap with luciferase and GFP fragments as needed for Gibson assembly method (forward: 5' TTT TGG CGT CTT CCA TGG TGG CGG TTG TGG CCA TAT TAT CAT CGT GTT TTT CAA AGG AAA ACC ACG TCC 3'; reverse: 5' ACT CTC GGC ATG GAC GAG CTG TAC AAG TAA AGC GGC CGG CCG CCA GCA CAG TGG TCG AAA 3'). All PCRs were performed using Q5 High-Fidelity DNA Polymerase according to the manufacturer's protocol. Amplified fragments were isolated from agarose gel after electrophoresis using Gel-Out kit (A&A Biotechnology) and subjected to Gibson Assembly using Gibson Assembly Master Mix (New England Biolabs) according to manufacturer's protocol. Reaction mixture was subsequently used to transform MAX Efficiency DH5α competent bacteria (ThermoFisher Scientific) upon which bacterial colonies were picked, amplified, and subjected to plasmid isolation with Plasmid Mini kit (A&A Biotechnology). Obtained plasmids were verified for the presence of luciferase coding sequence and further amplified.

In the next step HO-1 and SDF-1α coding sequences were introduced into LeGO-iLG2 plasmid. The former was excised from previously constructed LeGO-HO-1-iG2 plasmid [2] using BamHI and EcoRI restriction enzymes. The same endonucleases were used to cut LeGO-iLG2 plasmid and both fragments were ligated. SDF-1α coding sequence was amplified using primers introducing BamHI and EcoRI recognition sites (forward: 5' CTA CGG ATC CGC CAC CAT GAA CGC CAA GGT CGT GGT C 3'; reverse: 5' CTA CGA ATT CAC TTA CTT GTT TAA AGC TTT CTC 3'), isolated from agarose gel and digested with EcoRI and BamHI restriction enzymes. Obtained sequence was ligated with LeGO-iLG2 plasmid cut with the same endonucleases. The organization of vectors used for transduction of ADSC cells is shown in Fig.1A

All ligation were performed using T4 DNA Ligase (New England Biolabs) followed by transformation of MAX Efficiency DH5α competent bacteria, inoculation of LB medium (BioShop) and plasmid isolation with Plasmid Midi AX kit (A&A Biotechnology).

To obtain genetically-modified hiPSC already constructed transgenes (IRES-Luc-GFP, SDF-1α-IRES-Luc-GFP and HO-1-IRES-Luc-GFP) were transferred into a different plasmid backbone to replace viral-derived SFFV promoter, which we observed was prone to silencing in pluripotent stem cells (data not shown) into ubiquitin C (UbC) promoter. For that purpose LeGO-iLG2, LeGO-SDF-1α-iLG2, LeGO-HO-1-iLG2 and FUGW (Addgene#14883 [3]) plasmids were digested with BamHI and BspEI restriction enzymes. Transgene and FUW plasmid backbone fragments were then isolated from agarose gel and ligated. The organization of vectors used for transduction of hiPSC cells is shown in Fig.4A

2.2. Production of lentiviral vectors and transduction of cells

VSV-G pseudotyped lentiviral vectors were produced as previously described [4]. Briefly, 7x10^6 of 293T cells were seeded on 10 cm plates and transfected with 20 µg of transgene containing plasmid as well as 15 µg of psPAX2 and 6 µg of pMD2G plasmids using polyethylenimine (linear, MW 25000, Polysicences) as transfection reagent. Vector-containing medium was collected after 48h and 72h, centrifuged (200 × g, 5 min, RT) and filtered through 0.45 µm low protein binding filter.

To transduce ADSC cells were seeded on 15 cm plate and cultured for 24h in 15 ml of vector-containing medium supplemented with 5 µg/ml polybrene. Fresh ADSC medium was then added and cells were sorted based on GFP expression .

To transduce hiPSC lentiviral vectors were additionally purified by centrifugation of vector-containing medium (23000 × g, 3h, 4°C) to avoid the influence of 293T culture medium on hiPSC growth and pluripotency. 1/10 of the vector concentrate was added to hiPSC cultured in 12-well plate in E8 medium supplemented with 5 µg/ml polybrene. The medium was replaced with 1 ml of fresh E8 after 24 h and GFP-expressing cells were sorted after further expansion.

2.3. Cells sorting

Transduced ADSC were harvested using 0,25% trypsin/EDTA solution, centrifuged (200 × g, 5 min, RT), washed with PBS and resuspended in PBS supplemented with 2% FBS. GFP-expressing cells were purified using MoFlo XDP cell sorter (Beckman Coulter).

Transduced hiPSC were harvested using 0,5 mM EDTA solution, centrifuged (200 × g, 5 min, RT), washed with PBS and resuspended in PBS supplemented with 10 µM Y27632. GFP-expressing were purified using MoFlo XDP cell sorter.

2.4. Flow cytometry analysis

To assess cardiac differentiation efficiency, flow cytometry analysis of cardiac troponin T (cTnT)-positive cells was performed. For that purpose, cells were collected using Multi Tissue Dissociation kit 3 as described above, washed with PBS and stained with mouse anti-cTnT antibody (1:1000, ThermoFisher Scientific, clone: 13-11) using BD IntraSure Kit (BD Biosciences) according to manufacturer's protocol. Goat-anti mouse AlexaFluor568 (1:400, ThermoFisher Scientific) was used as secondary antibody and the signal was collected on LSRFortessa cytometer (BD Biosciences).

2.5. Luciferase activity assay

To confirm luciferase expression in genetically-modified ADSC luciferase activity assay was performed. For that purpose, proteins were isolated from the cells as described above. 5 µl of protein-containing lysates were placed in transparent 96-well plate and mixed with 100 µl of Luciferase Assay Reagent (Luciferase Assay System, Promega). Luminescence was measured using Tecan Infinite 200 PRO microplate reader and normalized to protein concentration.

2.6. Western blotting

To confirm HO-1 overexpression in genetically-modified ADSC and hiPSC Western blot analysis was performed as described previously [2]. Cells were washed and collected in PBS, centrifuged (10 min, 8000 × g, 4°C), resuspended in lysis solution (1% Triton X-100 in PBS) supplemented with 1x Complete protease inhibitor cocktail (Roche). After 30 min incubation on ice samples were centrifuged (10 min, 8000 × g, 10 min, 4°C) and proteins-containing lysate was transferred to new tubes and subjected to protein concentration analysis using bicinchoninic acid assay (BCA, Sigma-Aldrich). Samples containing 10 µg of proteins were subjected to 12% SDS-polyacrylamide gel electrophoresis (SDS-PAGE), transferred to nitrocellulose membrane (wet transfer, 30 V, overnight, 4°C), blocked in 5% fat-free powdered milk in PBS with 0.1% Tween20 for 2h at RT and incubated overnight at 4°C with primary antibodies: 1:1000 rabbit anti-HO-1 (Enzo, polyclonal) and 1:1000 mouse anti-α tubulin (Calbiochem, clone: DM1A) diluted in blocking buffer. After 5 washing steps with 0.1% Tween20 in PBS (PBST) membranes were incubated for 1h, RT with secondary antibodies: 1:10000 anti-rabbit HRP-linked (Cell Signaling) and 1:10000 anti-mouse HRP-linked (BD Pharmingen) diluted in blocking buffer. After another 5 washing step in PBST membranes were incubated for 5 min with SuperSignal West Pico Chemiluminescent Substrate Kit (Pierce Chemical) and chemiluminescent signal was developed on photographic films.

2.7. ELISA assay

To confirm SDF-1α overexpression in genetically-modified ADSC and hiPSC ELISA assay was performed using 50 µl of media collected from the cultured cells and Human CXCL12/SDF-1 alpha Quantikine ELISA kit according to the manufacturer's protocol. Concentration of secreted SDF-1α was normalized to the concentration of proteins isolated from the cells as described above

To confirm SDF-1α secretion from hiPSC-CM-SDF-1-Luc-CM which retained in murine myocardium, 42 days after MI induction and cell administration blood samples were collected from animals and centrifuged (800 × g, 10 min). Plasma was transferred to new Eppendorf tubes and stored at -80°C). To detect human SDF-1α Human CXCL12/SDF-1 DuoSet ELISA (R&D Systems) was used according to manufacturer's protocol. 50 µl of plasma samples were taken into analysis.

2.8. Spontaneous *in vitro* differentiation of hiPSC via embryoid bodies (EBs)

To assess differentiation potential of hiPSC line used in the study cells were harvested in E8 supplemented with 10 µM Y27632 (Abcam) Rho kinase inhibitor, centrifuged and resuspended in Essential 6 medium (E6, ThermoFisher Scientific) supplemented with 10 µM Y27632. $3x10^3$ cells/well were then seeded on non-adherent U-shaped 96 well plate (Corning). After 48h medium was replaced with E6 and on day 5 of differentiation formed EBs were transferred onto Geltrex-coated 48-well plate in fresh E6. Cells outgrowing from attached EBs were further cultured for 14 days in E6 changed every third day and subsequently subjected to immunofluorescent analysis of markers characteristic for endoderm, mesoderm and ectoderm.

2.9. Immunofluorescent analysis

To confirm expression of pluripotency markers in hiPSC line used in the study, cardiac markers in hiPSC-CM generated from parental hiPSC line as well as expression of three germ layers' markers in spontaneously *in vitro* differentiated hiPSC immunofluorescent analysis was performed as described previously [2]. Briefly, hiPSC were seeded on Geltrex-coated 48-well plate and cultured in E8 for three days after which the medium was removed, cells were washed with PBS and fixed in 4% paraformaldehyde (PFA, diluted in PBS; Sigma-Aldrich), 15 min, RT. Similarly, medium was removed from differentiating EBs, cells were washed with PBS and fixed in 4% PFA. After subsequent washing with PBS, cells were permeabilized with 0,1% Triton X-100 diluted in PBS, 15 min, RT, washed 3 times with PBS and blocked with 4% bovine serum albumin (blocking buffer; Sigma-Aldrich) diluted in PBS, 1h, RT. After blocking, cells were incubated with primary antibodies diluted in blocking buffer [for hiPSC-CM staining, 1% donkey serum (Sigma-Aldrich) in PBS was used to dilute antibodies] at 4°C, overnight: hiPSC – 1:200 goat anti-OCT4 (Santa Cruz Biotechnology, polyclonal), 1:100 rabbit anti-NANOG (Abcam, polyclonal), 1:100 mouse anti-SSEA4 (Merck Millipore, clone: MC-813-70), 1:100 mouse anti-TRA-1-60 (Merck Millipore, clone: TRA-1-60) and 1:100 mouse anti-TRA-1-81 (Merck Millipore, clone: TRA-1-81), hiPSC-CM – 1:100 mouse anti-α-actinin (Sigma-Aldrich, clone EA-53), 1:200 mouse anti-cardiac troponin T (ThermoFisher Scientific, clone 13-11) and 1:200 goat anti-NKX2.5 (Santa Cruz Biotechnology, polyclonal); differentiating EBs – 1:200 goat anti-GATA4 (Santa Cruz Biotechnology, polyclonal), 1:100 mouse anti-Alpha-fetoprotein (Santa Cruz Biotechnology, polyclonal) and 1:1000 rabbit anti-Neurofilament heavy polypeptide (Abcam, polyclonal). After washing (5 times in PBS) cells were incubated with secondary antibodies diluted in blocking buffer for 1h, RT: 1:400 AlexaFluor568 rabbit anti-goat, 1:400 AlexaFluor488 goat anti-rabbit and 1:400 AlexaFluor488 goat anti-mouse, AlexaFluor488 donkey anti-mouse and AlexaFluor568 donkey anti-goat (all from ThermoFisher Scientific). Subsequently, cells were incubated for 10 min with 1 µg/ml Hoechst33342 or 0,2 µg/ml DAPI (Sigma-Aldrich) diluted in PBS and washed 4 times in PBS. Stained hiPSC were visualized under Nikon Eclipse TS100 fluorescent microscope whereas hiPSC-CM and spontaneously differentiated hiPSC under Carl Zeiss LSM-510 meta laser scanning confocal microscope.

2.10. Alkaline Phosphatase activity assay

To confirm activity of alkaline phosphatase in hiPSC used in the study cells were seeded on Geltrex-coated 48-well plate, cultured for three days and assayed using Leukocyte Alkaline Phosphatase Kit (Sigma-Aldrich) according to manufacturer's protocol.

Supplementary References

1. Weber K, Bartsch U, Stocking C, Fehse B. A multicolor panel of novel lentiviral “gene ontology” (LeGO) vectors for functional gene analysis. Mol Ther J Am Soc Gene Ther. 2008;16:698–706.

2. Stepniewski J, Pacholczak T, Skrzypczyk A, Ciesla M, Szade A, Szade K, et al. Heme oxygenase-1 affects generation and spontaneous cardiac differentiation of induced pluripotent stem cells. IUBMB Life. 2018;70:129–42.

3. Lois C, Hong EJ, Pease S, Brown EJ, Baltimore D. Germline transmission and tissue-specific expression of transgenes delivered by lentiviral vectors. Science. 2002;295:868–72.

4. Stepniewski J, Kachamakova-Trojanowska N, Ogrocki D, Szopa M, Matlok M, Beilharz M, et al. Induced pluripotent stem cells as a model for diabetes investigation. Sci Rep. Nature Publishing Group; 2015;5:1–14.

Supplementary Figure 1

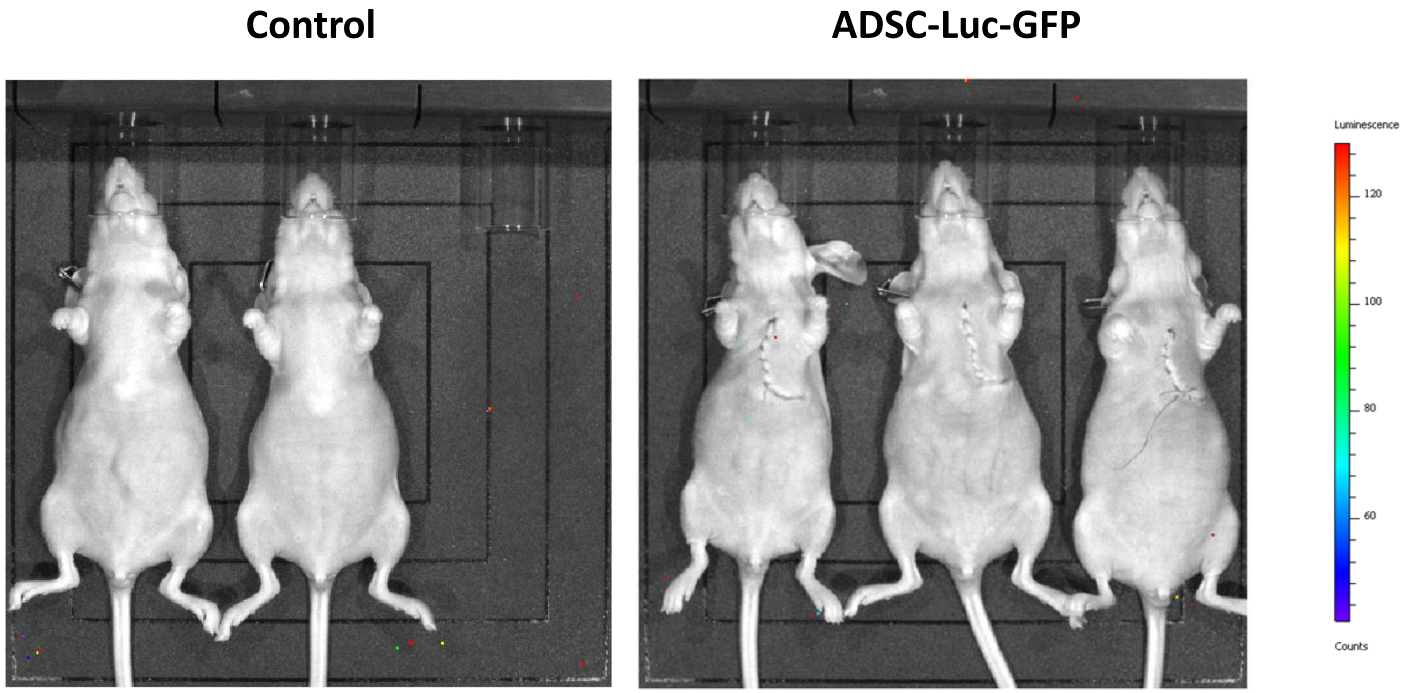

Supplementary Figure 2

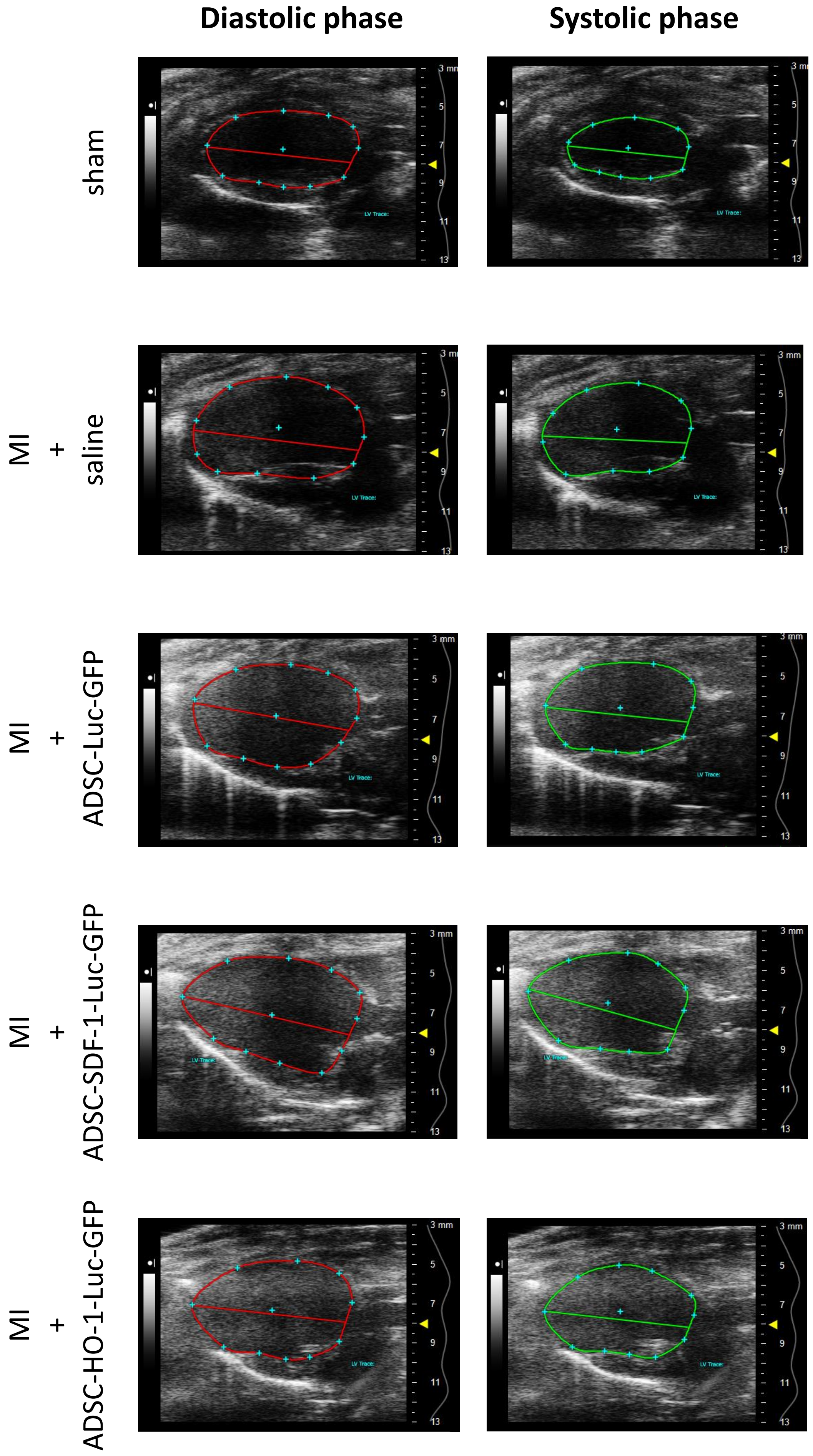

Supplementary Figure 3

a

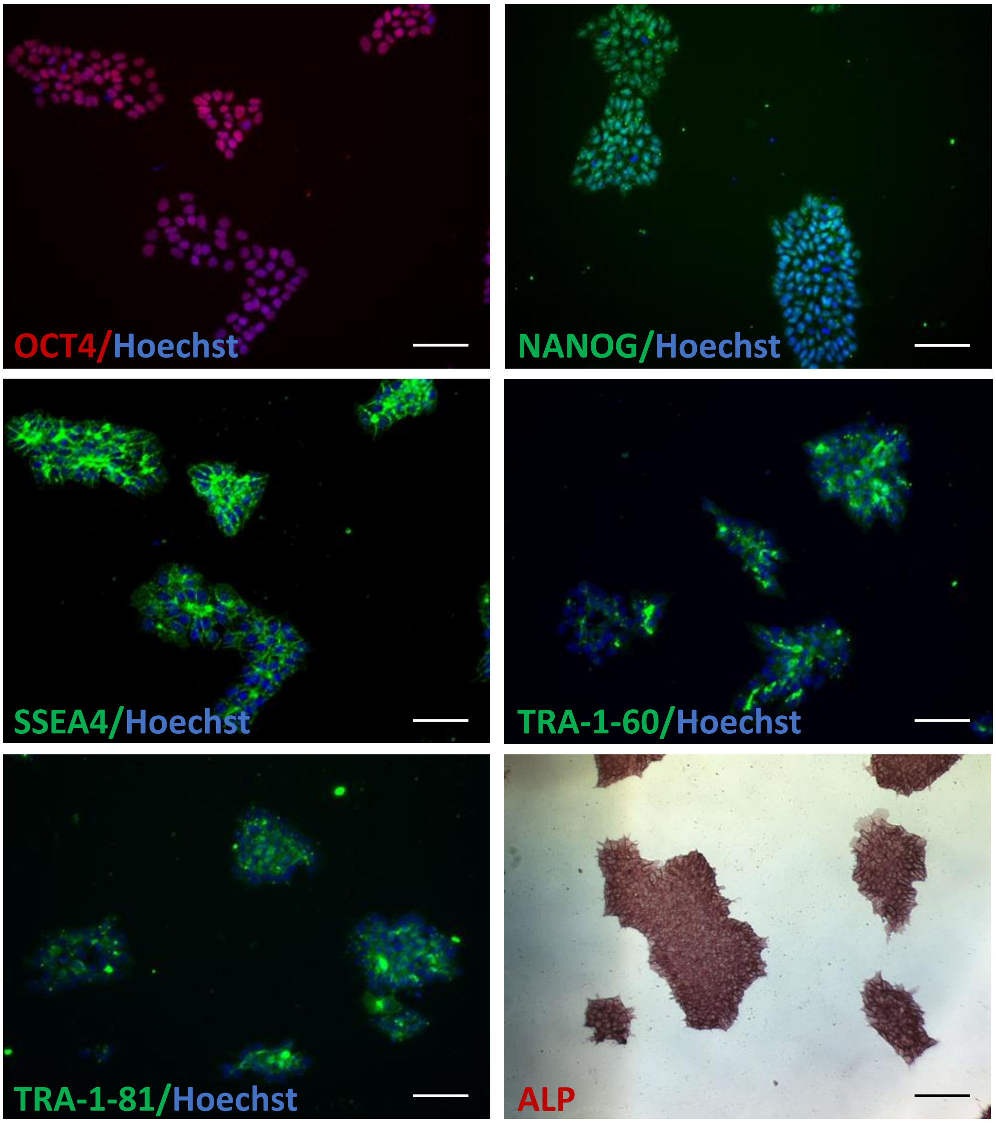

b

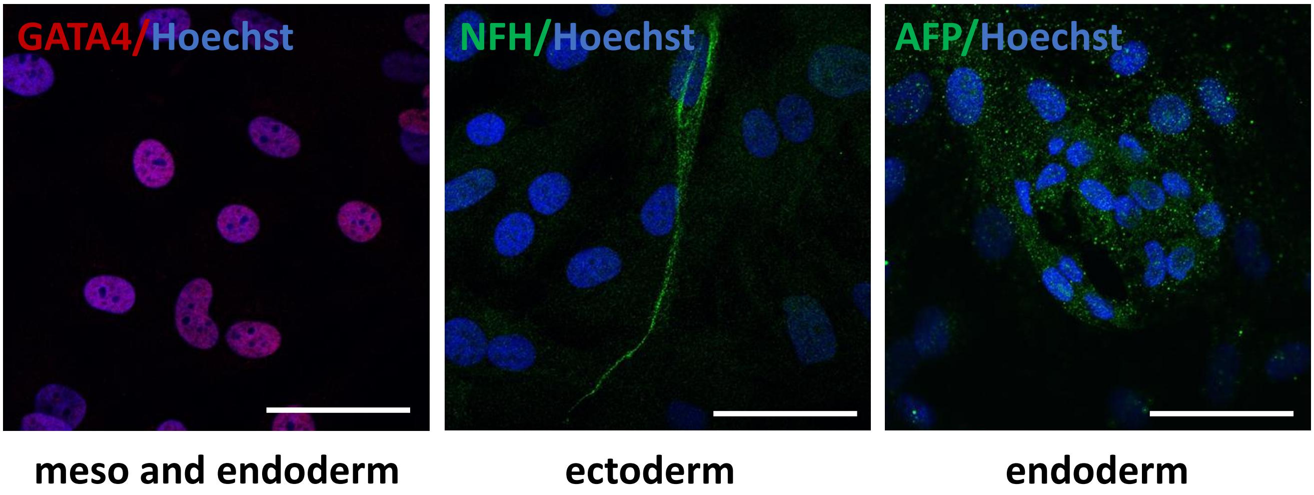

Supplementary Figure 4

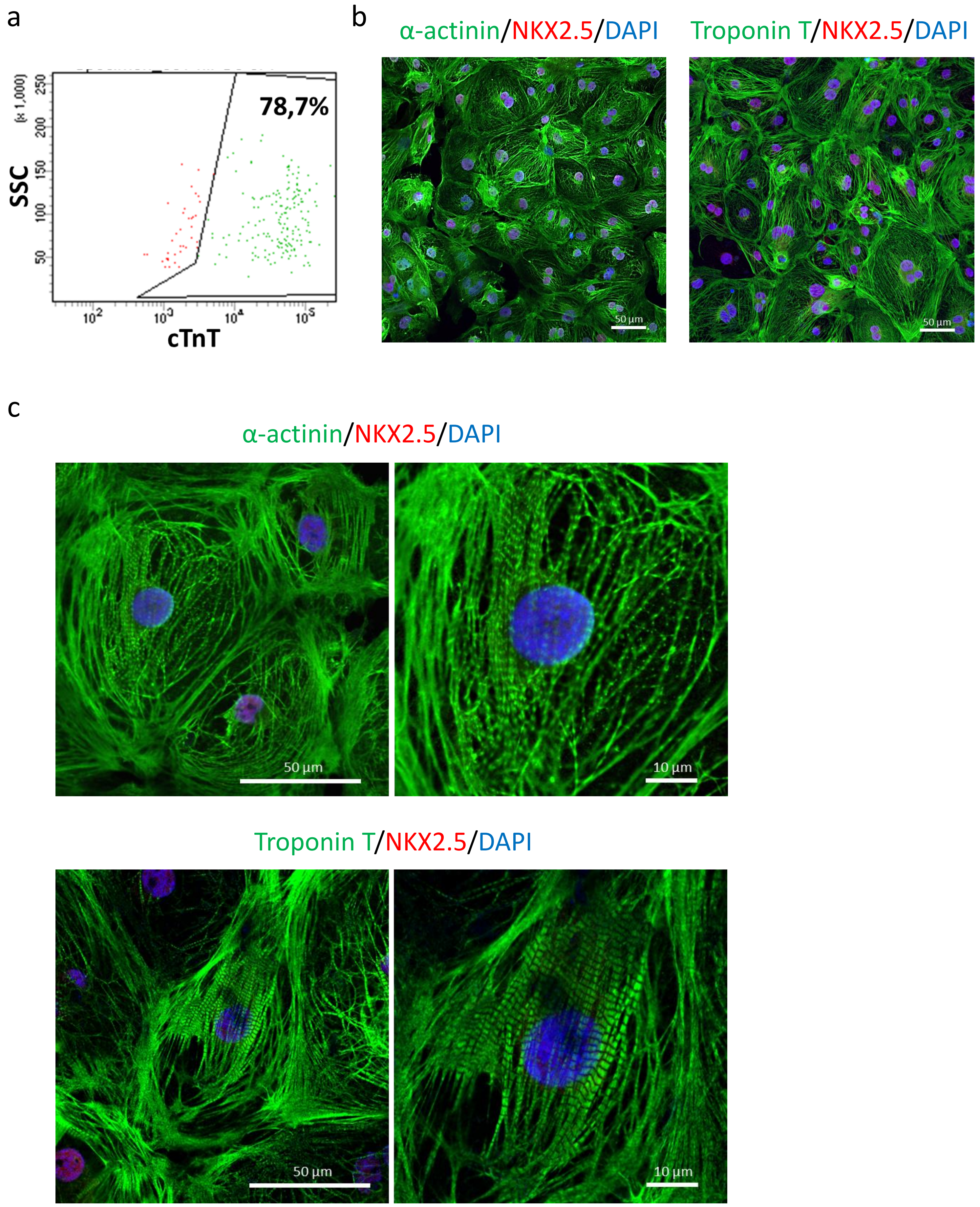

Supplementary Figure 5

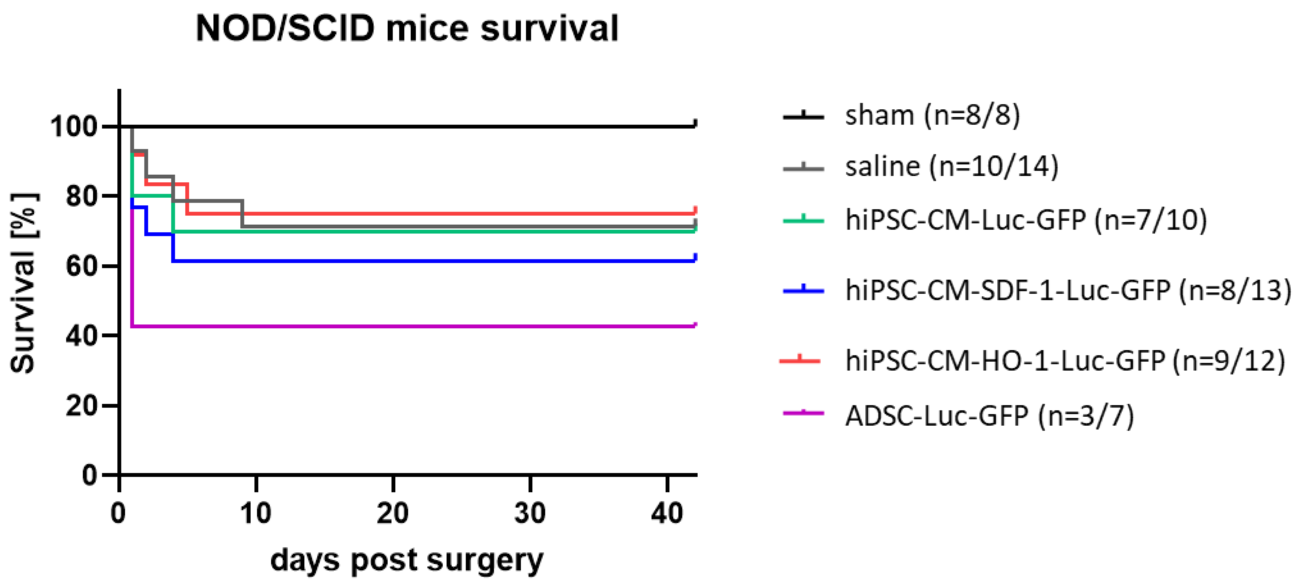

Supplementary Figure 6

a

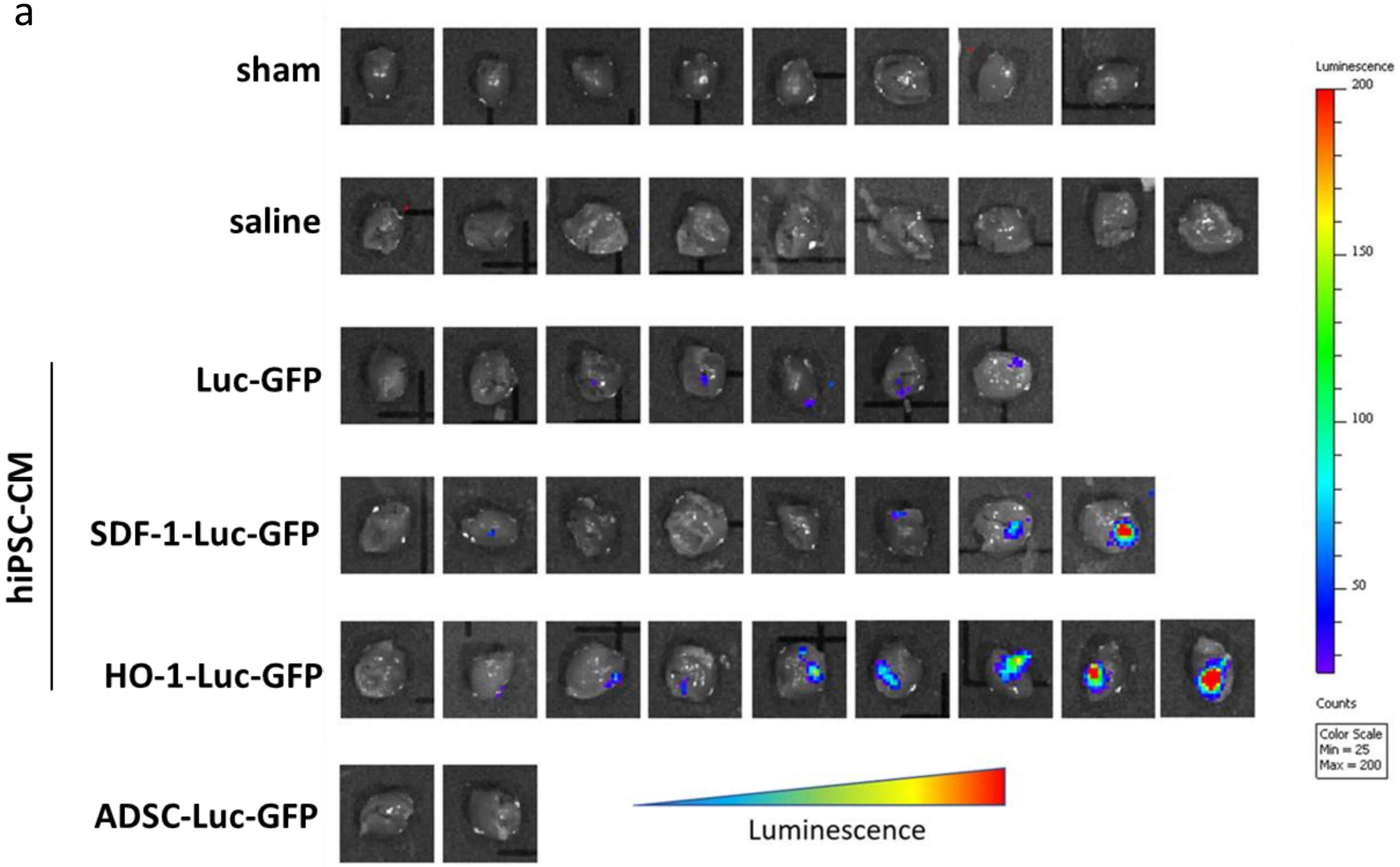

b

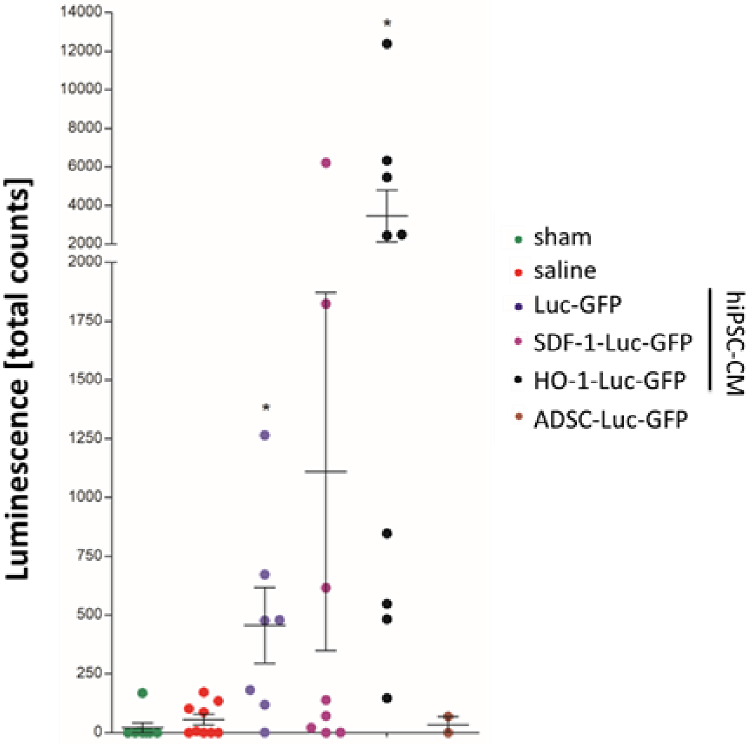

Supplementary Figure 7

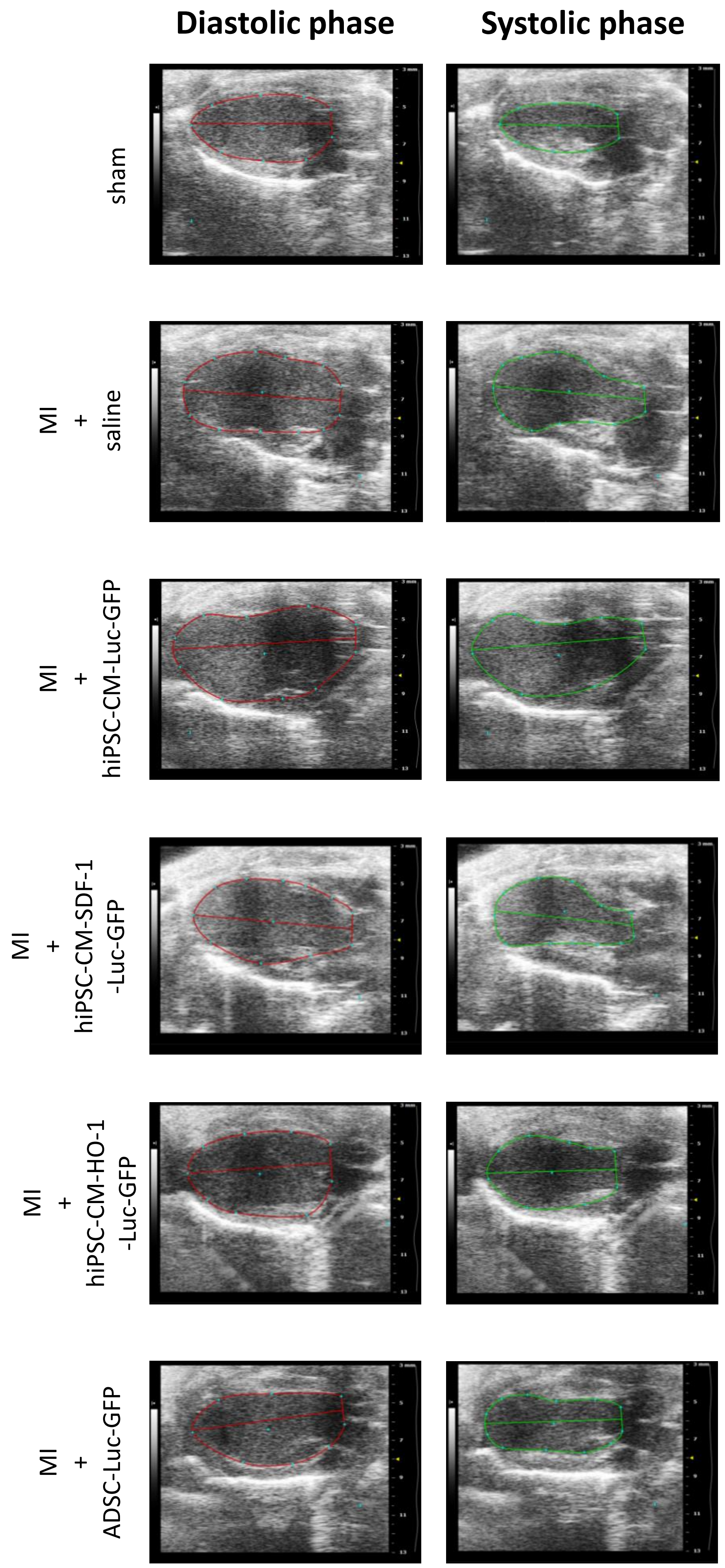

Supplementary Figure 8

hiPSC-CM-Luc-GFP

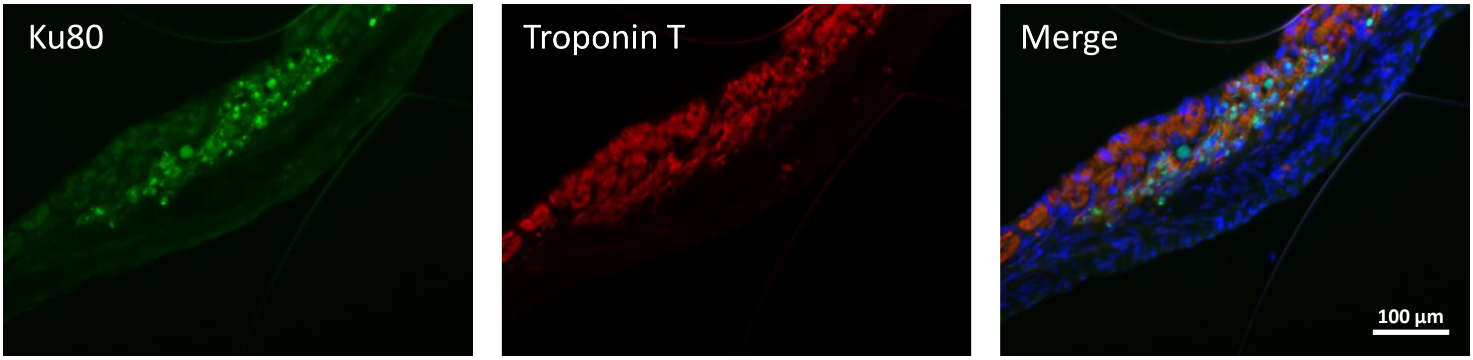

hiPSC-CM-SDF-1-Luc-GFP

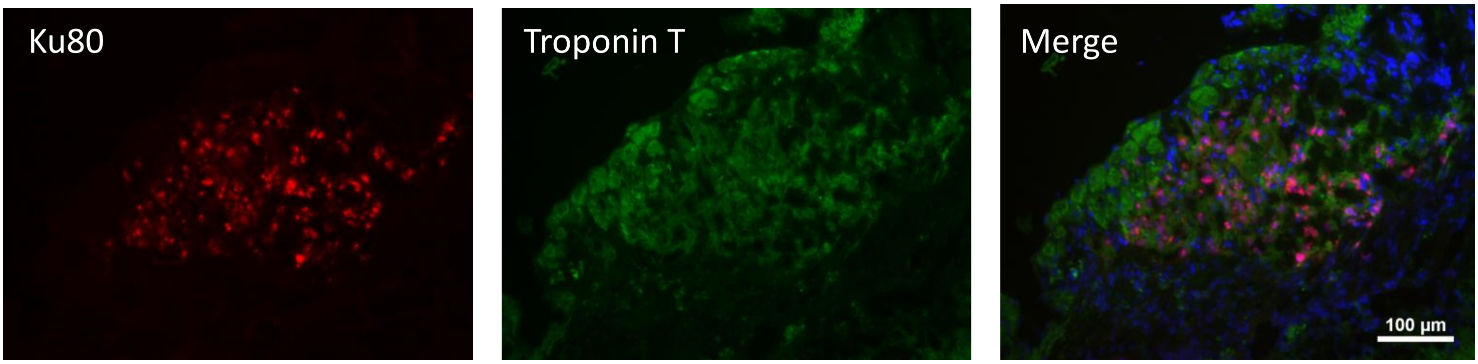

hiPSC-CM-HO-1-Luc-GFP

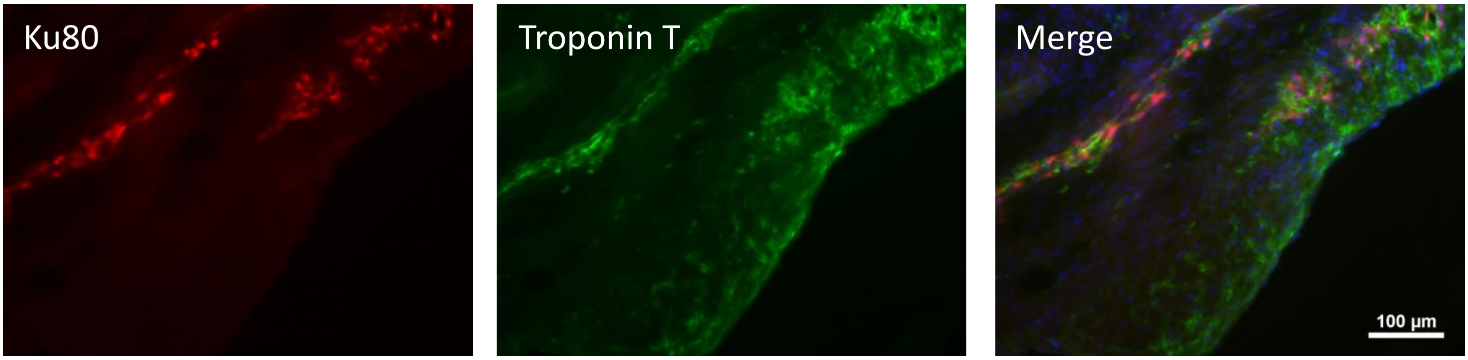

Supplementary Figure 9

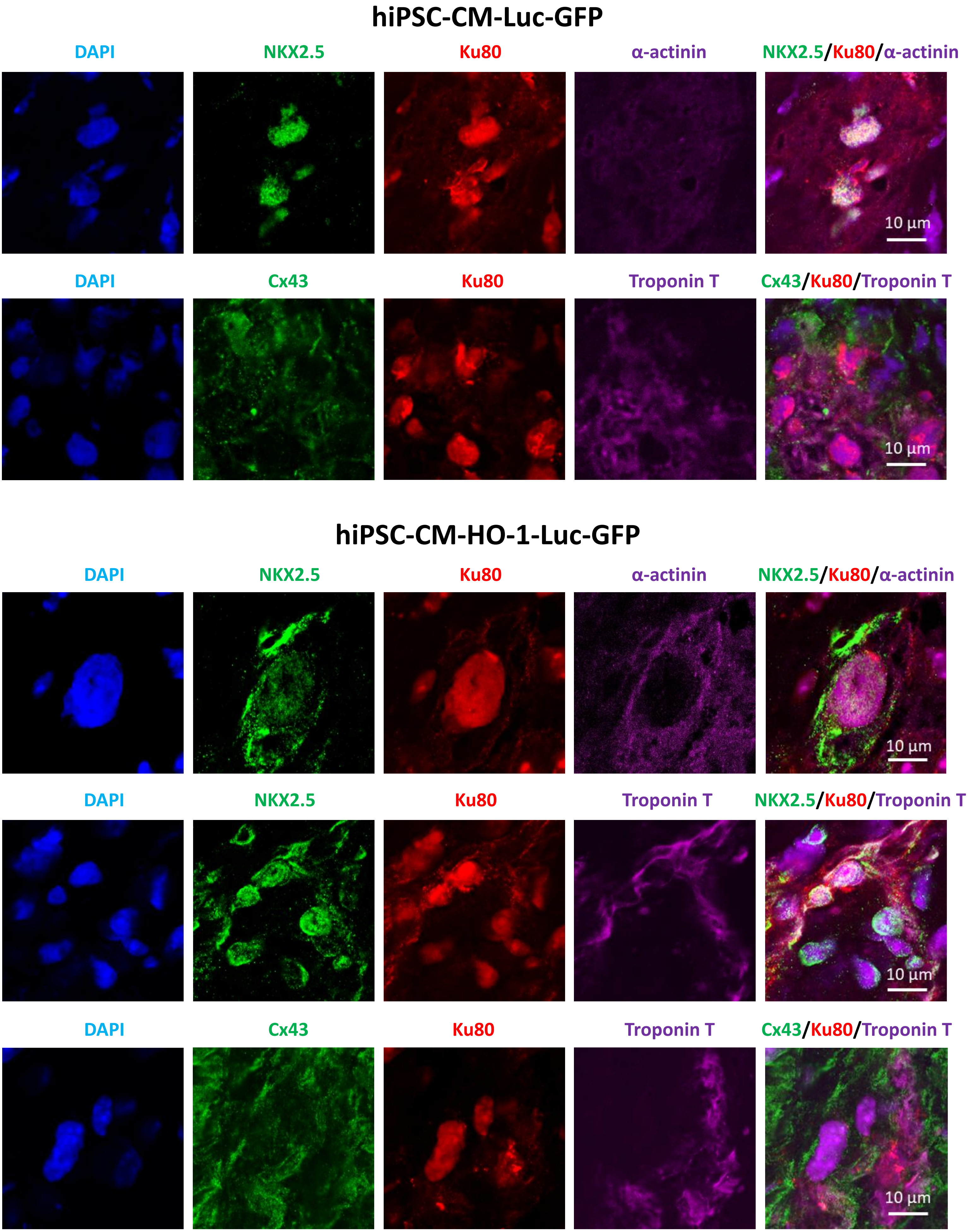

Supplement: Supplementary file 1 [file biomedicines-08-00578-s001.pdf]
